# Anti-Inflammatory Peroxidized Chlorahololide-Type Dimers Are Artifacts of Shizukaol-Type Dimers: From Phenomena Discovery and Confirmation to Potential Underlying Mechanism

**DOI:** 10.3390/molecules29040909

**Published:** 2024-02-19

**Authors:** Xiu-Wen Yin, Ming Zhang, Lan Wu, Fu-Cai Ren, Fu-Rong Yang, Xiang-Dong Pu, Zhi-Jun Zhang, Chuan-Pu Shen

**Affiliations:** 1 Anhui Provincial Laboratory of Inflammatory and Immunity Disease, Anhui Institute of Innovative Drugs, School of Pharmacy, Anhui Medical University, Hefei 230032, China; 2 School of Pharmacy, Hubei University of Science and Technology, Xianning 437100, China

**Keywords:** *Chloranthus*, sesquiterpene dimers, transformation, quantum chemical calculation, anti-inflammatory activity

## Abstract

In our research on naturally occurring sesquiterpenes, eight shizukaol-type dimers, one chlorahololide-type dimer, and one sarcanolide-type dimer were isolated from the roots of *Chloranthus fortunei.* As the project was implemented, we accidentally discovered that shizukaol-type dimers can be converted into peroxidized chlorahololide-type dimers. This potential change was discovered after simulations of the changes in corresponding shizukaols showed that three peroxide products were generated (**1–3**), indicating that peroxidation reactions occurred. HPLC-HR-MS analysis results obtained for the shizukaol derivatives further demonstrate that the reaction occurred, and the type of substituent of small organic ester moieties at positions C-15’ and C-13’ of unit B were not decisively related to the reaction. Quantum chemical calculations of the mode dimer further demonstrated this phenomenon. The highest occupied molecular orbital (HOMO)–lowest unoccupied molecular orbital (LUMO) energy of the precursor and production revealed the advantageous yield of 4*β*-hydroperoxyl production. Additionally, the potential reaction mechanism was speculated and validated using the free energy in the reaction which successfully explained the feasibility of the reaction. Finally, the anti-inflammatory activity of the precursors and products was evaluated, and the products of peroxidation showed better anti-inflammatory activity.

## 1. Introduction

**Figure 1:**
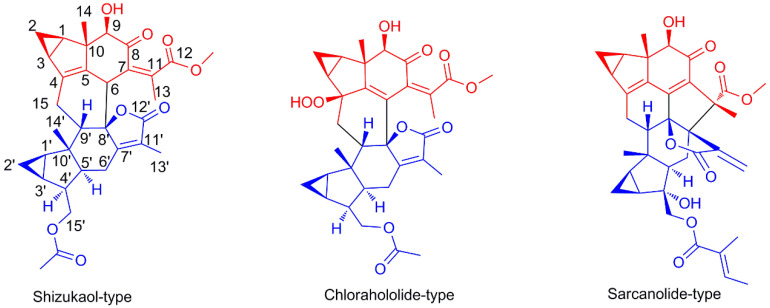
The main types of sesquiterpene dimer isolated from *Chloranthus* plants; red for unit A, blue for unit B.

The sesquiterpenoid dimer is a novel compound built by two sesquiterpene blocks via Diels–Alder addition or free radical reaction [1,2]. Due to their structural complexity, these compounds have attracted the attention of many natural product researchers. Among them, the sesquiterpene dimer obtained from *Chloranthus* plants is mainly composed of two lindenane-type sesquiterpenes molecules, which are obtained from Diels–Alder reactions [3]. To date, about 300 dimers have been isolated from this genus. Among them, three main types of dimers have been discovered, namely shizukaol-type, chlorahololide-type, and sarcanolide-type dimers (Figure 1). Shizukaol-type dimers were the earliest type found and the most widely reported. In addition to their complex structure, the sesquiterpene dimers displayed a wide range of biological activities, such as anti-inflammatory activity, antitumor activity, antimicrobial activity, and neuroprotective activity. These important characteristics have led to a high level of enthusiasm for research on these compounds [4,5,6].

Lindane-type sesquiterpene is characterized by cyclopropane with a strong ring strain at C1-C3. In the shizukaol-type sesquiterpene molecule, the positions of the cyclopropane (C1-C3) and double bond (C4-C5) are very close in Unit A. From an organic chemistry perspective, this structure is very unstable. The bridgehead double bond at C4-C5 becomes unstable under the tension of cyclopropane [7]. Thus, the stability of the compounds is questionable. Based on these properties, we speculate that the compounds are unstable.

The spontaneous transformation of natural products is an unusual phenomenon, and some examples of these reactions include keto-enol tautomerism, keto-amide tautomerism, and the ring opening and closing of *α*, *β*-unsaturated-*γ*-hydroxyl-*γ*-lactone [8,9,10,11,12,13,14]. In addition, the reactions of neighboring groups within molecules could cause spontaneous transformation in compounds. Cao et al. performed the Michael addition within withanolide molecules, a kind of characteristic constituent of the genus *Withania*, to generate a mixture containing artifacts and the original compound [15]. In response to these changes, Wu et al. developed a method to prevent this reaction from occurring, thereby obtaining a series of purified products [16]. The process of investigating these compounds, from discovering a phenomenon to explaining and utilizing it, is very enjoyable.

As part of our ongoing research on the chemical constituents of *Chloranthus* plants, sesquiterpenes were directionally isolated with UV guidance from the roots of *Chloranthus fortunei* (A. Gray) Solms-Laub. As a result, eight shizukaol-type dimers, four chlorahololide-type dimers, and one sarcanolide-type dimer were isolated. During the investigation of shizukaol-type sesquiterpene dimers, the compounds were found to be peroxidized and produced a new class of sesquiterpene dimers. This phenomenon has not been reported or taken seriously in the past. In this article, the phenomenon, transformation mechanism, and changes in the biological activity of dimers after spontaneous peroxidation are reported first.

## 2. Results and Discussion

**Figure 2:**
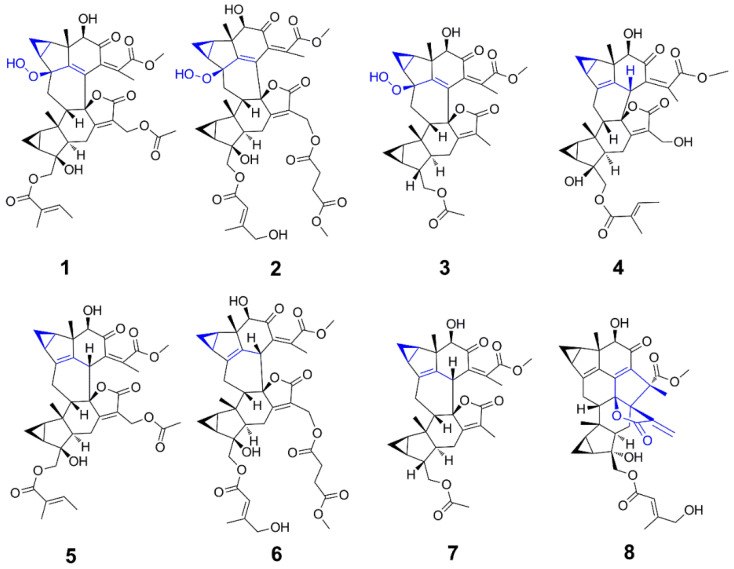
The representative compounds isolated from the roots of *Chloranthus fortunei.*

The roots of *Chloranthus fortunei* (15.0 kg) were powdered and extracted to afford a residue. After the partition, the EtoAc-soluble part was investigated under the guidance of a UV spectrum obtained from HPLC-DAD for the directional separation of lindenane-type sesquiterpenes. As a result, ten known compounds (Figure 2) were isolated and elucidated as shizukaol C (**4**) [17], chlorahololide D (**5**) [18], chololactone F (**6**) [19], shizukaol E (**7**) [20], fortulactone A (**8**) [21], shizukaol D (**9**) [17], fortunilide C (**10**) [22], 13’-dehydroxylation-sarcandrolide J (**11**) [23], sarglabolide J (**12**) [19], and chlorahololide B (**13**) [2] by a careful analysis of their NMR data and a comparison with the reported data in the literature. Up to this point, we have analyzed the planar and relative structures of the compounds. However, the accuracy of the structural analysis of the key compounds does have a significant impact on the results of this article. Therefore, the absolute configurations of the key compounds **4–6** were investigated. In the ECD of **4–6** (in Supporting Information S4), a right-handed helicity of two coupling chromophores determined that the configuration of C-8’ of compounds **4–6** were all *R* by the CD exciton chirality method. Furthermore, the absolute configurations of these compounds (**4–6**) were resolved with the help of relative configuration.

Due to the long straight-line distance of approximately one thousand kilometers between the location of the nuclear magnetic resonance (NMR) testing (Guilin, Guangxi) and the laboratory (Hefei, Anhui), much time passed before the compounds were analyzed. During this process, the samples were exposed to light and oxygen. After compound testing, we discovered a terrible phenomenon. As analyzed by high-performance liquid chromatography (HPLC) under the same conditions as purity testing, all shizukaol-type dimers seemed to decay after the long-term NMR test. However, before the samples were sent, purity tests were conducted on each compound using HPLC, and the results showed all compounds were pure enough to be tested. These phenomena suggested that the shizukaols may be unstable. The previous purity HPLC results were therefore re-examined, and one or more tiny peaks could be found in the peak areas with extremely early retention times in the chromatogram of each purified compound. The chromatograms used for validation after compound purification were very similar to those obtained after nuclear magnetic testing. The only difference was that these impurities became more numerous after long-term storage (approximately one month). These phenomena remind us that the compounds undergo conversion after purification.

**Figure 3:**
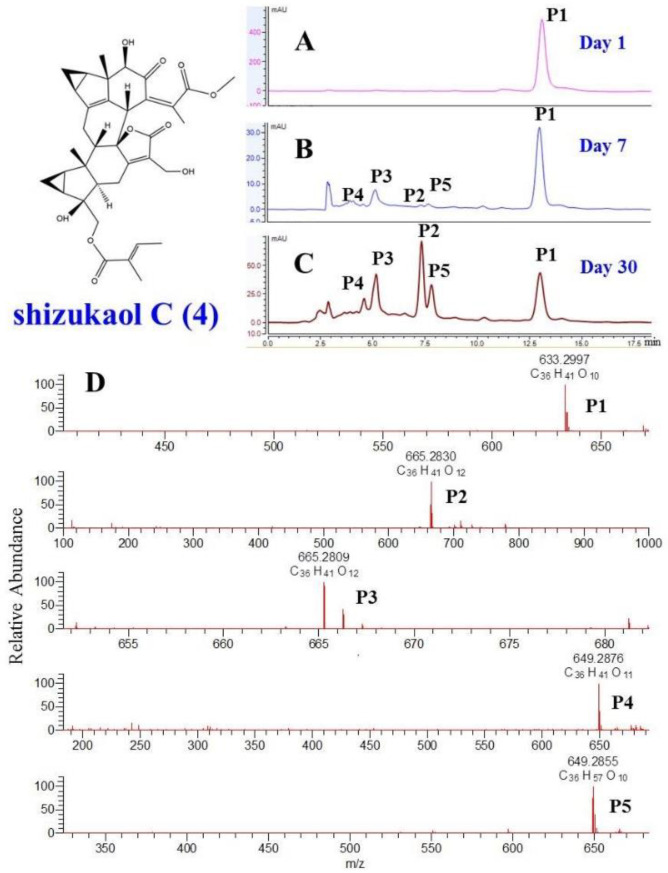
The confirmation of phenomena of the conversion of compound **4.** (**A**) HPLC chromatography of **4** after one day of the purification. (**B**) HPLC chromatography of **4** after one week of the purification. (**C**) HPLC chromatography of **4** after one month of the purification. (**D**) The result of the UPLC-HR-MS analysis of **4** after one month of the purification.

The primary tasks necessary to explain this phenomenon were to confirm it and clarify the structure of the products. We simulated the environment of compound isolation after repurification. All compounds were placed in a methanol aqueous solution at room temperature. During the process, we analyzed the solution after one day and one month later (Figure 3). The result showed that impurities appeared at the very beginning of the retention time in the HPLC chromatograph from the first day onward. To determine the structure of the impurities, we conducted high-concentration simulations on three shizukaol-type compounds in large amounts (>50.0 mg) and then purified the impurities using preparative HPLC. After the NMR tests, the three impurities were identified as spicachlorantin E (**1**) [24], fortunilide E (**2**) [22], and chololactone B (**3**) (Shen et al., 2017) [19] by the comparison of spectroscopic data with the literature, which contained known C-4 peroxidized chlorahololide-type dimers. Compounds **1**-**3** all have a typical 4*β*-peroxy hydroxyl group at C-4. To further address the absolute configurations of these compounds, the ECD (in Supporting Information S4) exciton chirality method was also utilized to characterize the absolute configuration of the key compounds. The absolute configurations at C-8’ of compounds **1–3** were determined as *R*, deduced from a right-handed helicity of two coupling chromophores, consistent with the absolute configuration of the substrate (**4–6**). To more intuitively verify whether these compounds are artifacts, the extract of the roots of *C. fortunei* were tested for an HPLC-HR-MS experiment. As a result, we did not find the presence of compounds **1**, **2**, and **3** in the crude extract, which prompted that peroxidized chlorahololide-type dimers may be generated by aforementioned reactions during purification and storage processes (Supporting Information Part I).

The three tested compounds were very similar to each other, except for the small organic ester substitution groups at positions C-15’ and C-13’ of unit B. To investigate the effect of substituents on the conversion of compounds, dimers with different substituents were tested. Due to the limited amount of precursors, the structure of the products could not be determined using the NMR method. The three impurities mentioned above were compared with the precursor compounds, and the molecular weight of the products and precursors differed by exactly two relative atomic masses for oxygen. Therefore, the MS analysis of HPLC-HR-MS could be very suitable for monitoring the occurrence of these reactions. For the former method, purified shizukaol C (**4**), chololactone F (**6**), shizukaol D (**9**), fortunilide C (**10**), 13’-dehydroxylation-sarcandrolide J (**11**), and sarglabolide J (**12**) were placed in solution to detect the transformation. After 30 days of simulations, we used HPLC-HR-MS to detect and analyze the solution.

Taking shizukaol C as an example (**4**) (Figure 3), after purification, it was immediately analyzed by HPLC-HR-MS, resulting in a retention time (*t*_R_) of 13.1 min when eluted with MeOH-H_2_O (60:40, *v*/*v*) and quasimolecular ion peaks at *m*/*z* 633.2997 [M − H]^−^ (P1), in accordance with the molecular formula C_36_H_40_O_10_. After careful observation, some tiny peaks appeared in the *t*_R_ 4.5–8.0 min interval of the chromatogram. At the end of the reaction for 30 days, all the tiny peaks became extremely prominent. Therefore, MS analysis of the solution was carried out. As a result of the MS analysis, the molecular formula of P2 was determined to be C_36_H_40_O_12_ based on the ion peak at *m*/*z* 665.2380 [M − H]^−^. Two more oxygen atoms were present in the molecular formula of P2 than in that of shizukaol C. Therefore, it can be concluded that P2 is a peroxide product of shizukaol C. Therefore, the changes in these compounds were mainly due to the peroxide reaction. On the basis of the data obtained herein for compounds **5–7**, it is reasonable to assume that all 4-hydroperoxyl lindanane dimers reported in the literature are most likely shizukaol-type dimer artifacts. The results also showed that the substituents at positions C-15’ and C-13’ of unit B had no effect on the changes in the compound. Peroxidation occurred in all the isolated shizukaols which then became impure (see Supporting Information Figures S2–S6).

**Figure 4:**
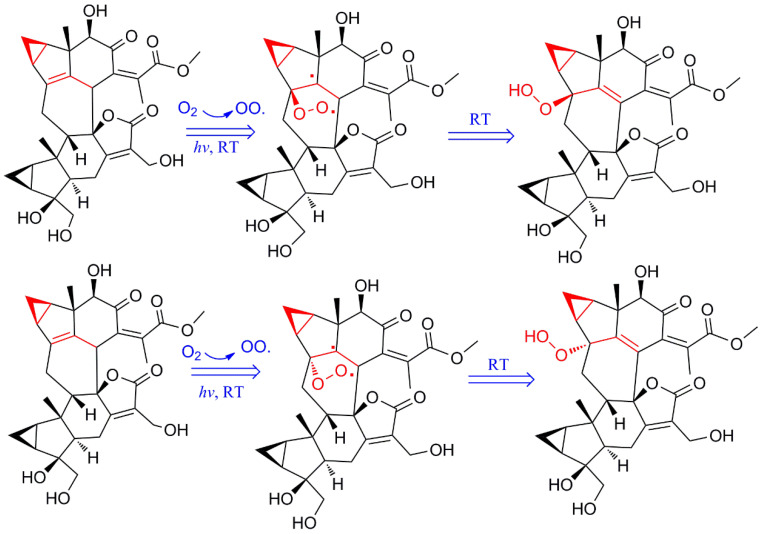
The proposed “decayed” pathway of the shizukaol model dimer.

Peroxidation natural products are a class of compounds with extremely high value. Among these compounds, the most important is artemisinin, a first-line antimalarial drug that was developed independently by scientists in China. Coincidentally, artemisinin is also a peroxidized sesquiterpene isolated from the natural resource *Artemisia annua* [25]. Therefore, although the peroxidation in this experiment is unfavorable for the separation of the original compound, it may generate higher-value products. Explaining this phenomenon is very important for obtaining these products more accurately and efficiently. In shizukaol molecules, the conjugation of cyclopropane and the C4-C5 double bond destabilize the structures under the tension of cyclopropane. Similar to the aforementioned results, the peroxide position of the isolated peroxidized chlorahololide-type dimers is located at C-4. Therefore, the generation of impurities is mainly caused by the instability of C4-C5 double bonds. The possible mechanism underlying the change was considered a free radical reaction (Figure 4). First, oxygen in the air produces oxygen free radicals under illumination. Then, reactions occur between oxygen radicals dissolved in solution and the original compounds. The C4-C5 double bond breakage produces two free radical fragments. Finally, the free radicals are annihilated in solution to produce the final products.

We conjecture that oxygen radicals can attack the C-4 position of the precursor in two ways, from the back or the front of the double bond. In addition, C-5 is a highly likely reaction site that can generate two kinds of products. However, the three purified peroxidized chlorahololide-type dimers were all 4*β*-hydroperoxyl in the C-4 position. Therefore, it can be speculated that the oxygen free radicals that attack from the 4*β* position may have lower energy or that the production is more stable. To confirm our conjecture, quantum chemical calculations were carried out to reveal the reasons behind the phenomenon. As the molecular weight increases, the difficulty of the calculation increases exponentially. Due to the complexity of the dimer molecule, the structure of these compounds must be simplified. Since the main change site is located at C-4, we only calculated C-4 peroxidation. The small organic acid esters at C-15’ and C-13’ of unit B cause no decisive effect on the changes in compounds. Therefore, in structural calculations, a mode dimer was simplified, as shown in Figure 4.

**Figure 5:**
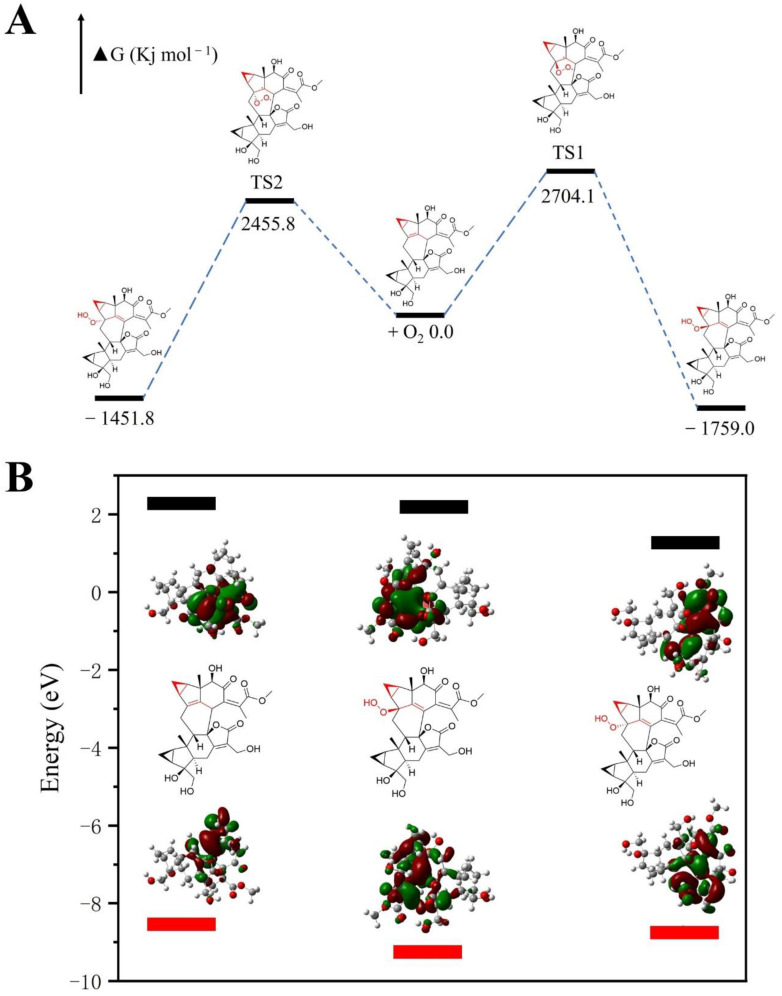
The results of free energy of the reaction (**A**) and the HOMO (black buds)-LUMO (red buds) energy of model dimer and production (**B**) calculated by using the DFT method. IM is the abbreviation for intermediate.

The density functional theory (DFT) method was employed to calculate the highest occupied molecular orbital (HOMO)–lowest unoccupied molecular orbital (LUMO) energy. The HOMO-LUMO energy gap is closely related to the stability of compounds. The larger the energy gap is, the more difficult it is for electronic transitions to occur, and the more stable molecules become. The results showed that the energy gap of *β*-hydroperoxyl production was higher than that of the original compounds; however, that of *α*-hydroperoxyl was lower than that of the precursor (Figure 5). Therefore, *α*-hydroperoxyl production is more unstable than *β*-hydroperoxyl production, and only *β*-hydroperoxyl products are obtained during the chemical transformation process. Furthermore, the HOMO energy of the precursor was higher than that of all products, demonstrating that the precursor was more easily oxidized. This can preliminarily reveal why these compounds can undergo peroxidation. Moreover, free energy can predict the reaction progress. DFT calculations can reliably calculate energy changes. We calculated the transformation process of the mode dimer using the DFT method, and the results showed that peroxidation at position C-4 can occur spontaneously in room-temperature solution (Figure 4). 4*β*-hydroperoxyl production is easier to obtain than 4*α*-hydroperoxyl production, which is consistent with the aforementioned simulation results.

**Figure 6:**
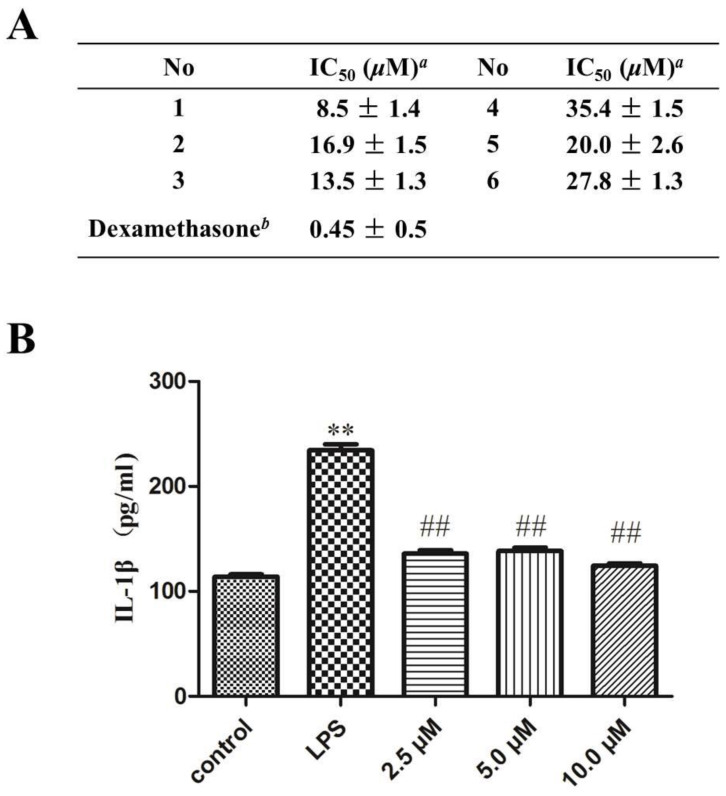
(**A**) Inhibitory effects of isolates on NO release in LPS-stimulated RAW 264.7 macrophages. *^a^* Represented mean ± SD from three independent experiments; *^b^* dexamethasone as positive control. (**B**) The inhibitory effects of the IL-1*β* in LPS-stimulated RAW 264.7 macrophages of **1**, ** *p* < 0.01 versus the control group, ^##^ *p* < 0.01 versus the LPS group. Data are expressed as means ± SD.

The roots of *Chloranthus fortunei* are used as a traditional Chinese medicine for the treatment of inflammatory diseases. Macrophages play an important role in the development and transformation of many inflammatory diseases [26]. LPS is an important component of the cell wall of Gram-negative bacteria, which can cause inflammation, shock, infection, etc. [27,28]. It induces RAW264.7 macrophages to secrete pro-inflammatory factors and produce inflammatory responses, which is a classic inflammatory cell model. After LPS treatment, macrophages migrate to a pro-inflammatory state, causing the inflammatory target mediator NO and inflammatory cytokine IL-1*β* to be significantly expressed, which will promote the inflammatory process [29,30,31]. This model has been widely applied in the evaluation of anti-inflammatory activity of natural products [32,33,34]. Thus, the key compounds of concern (**1–6**) were purified and quickly tested for their inhibitory effects on NO production in LPS-induced RAW 264.7 macrophages. As the results (Figure 6A) showed, the peroxidized chlorahololide-type dimers (**1–3**) displayed more potential effects than those of the shizukaol dimers (**4–6**), which may have resulted from the existence of the peroxide moiety. In LPS-stimulated RAW 264.7 macrophages, proinflammatory cytokines such as IL-1*β* play an important role in the occurrence and development of inflammation. Due to the limitation quantity available, only compound **1** was further tested for its inhibitory effects on the proinflammatory cytokine IL-1*β*. Compound **1** can effectively inhibit the expression of IL-1*β* (Figure 6B). Therefore, peroxidized chlorahololide-type dimers showed greater therapeutic potential for inflammatory diseases than shizukaol dimers and are worthy of our attention.

In this article, the decay of shizukaol-type dimers to peroxidized chlorahololide-type dimers is reported for the first time, and the research involved the discovery and confirmation of the phenomenon. These results also suggested that peroxidized chlorahololide-type dimers are highly likely artificial products which were determined by the results of HPLC-HR-MS and the chemical transformation. In addition, the mechanisms were comprehensively explained using chemical transformation and quantum chemistry calculations. *Chloranthus* plants are among the research hotspots of natural products. Many patents have been filed for some sesquiterpenes and their dimers as drug candidates. Therefore, the stability of these compounds deserves further attention, and developing drugs from these compounds would be a significant advancement. Compared to the shizukaol-type dimer, the peroxidized chlorahololide-type dimer exhibits a more stable structure and better bioactivity. However, when compound **4** was studied with HPLC-HR-MS, the results showed that the hydroperoxyl hydroxyl groups were reduced, as deduced from the loss of one oxygen atom. In order to gain a more comprehensive understanding of the stability of lindanane-type sesquiterpene dimers, preliminary research on the stability of the other types of dimers has been conducted, and the results show that these compounds are stable (Supporting Information Figures S33 and S34). Additionally, compared to the large presence of shizukaol-type dimers, which have been artificially synthesized [23], chlorahololide-type dimers are rare. The biological effect evaluation showed that peroxidized chlorahololide-type dimers exhibit more potential anti-inflammatory activity and better medicinal value. The results of this article will provide ideas for methods to artificially synthesize chlorahololide-type dimers. Further biological studies need to be conducted after the low yield of the obtained compounds is increased.

## 3. Experimental Section

### 3.1. General Experimental Procedures

NMR spectra were recorded on a Brucker Avance III 500 NMR instrument (^1^H: 500 MHz, ^13^C: 125 MHz, Brucker, Billerica, MA, USA), with TMS as internal standard. Mass spectra were recorded on a Dionex Ultimate 3000 UPLC-Q Exactive Plus (HR-ESI-MS) (Dionex, Sunnyvale, CA, USA). Silica gel (Qingdao Haiyang Chemical Co., Ltd., Qingdao, China) and RP-C18 (40–63 μm, Fuji, Chiryu, Japan) were used for column chromatography. Semipreparative HPLC was carried out using a Thermos N3000 series instrument (Thermos, Schaumburg, IL, USA) with an H&E RP-C18 column (10 × 200 mm) or a Shimadzu LC-20AR series instrument (Shimadzu, Kyoto, Japan) with a Shimpak RP-C_18_ column and an SPD-20A variable-wavelength detector (Shimadzu, Kyoto, Japan).

### 3.2. Plant Material

The roots of *Chloranthus fortunei* were collected from Guangxi Province, China, and identified by Xie Jin from Anhui Medical University. A voucher specimen (No. 20180520) was deposited in School of Pharmacy, Anhui Medical University.

### 3.3. Isolation and Purification

The roots of *Chloranthus fortunei* (15.0 kg) were powdered and extracted at room temperature by using ethanol/H_2_O (95/5, *v*/*v*) three times to afford a residue (696.0 g) after the solution was removed. The residue was suspended in water and partitioned successively by petrol ether (PE) and ethyl acetate (EtOAc) to obtain PE-soluble (99.8 g) and EtOAc-soluble (476.9 g) fractions. The EtOAc-soluble fractions were subjected to silica gel column chromatography (CC) gradient elution by CHCl_3_/CH_3_OH (from 100:1 to 5:1) to afford seven subfractions (Fr. EA-EG). Fr. EA (7.78 g) was subjected to silica gel CC eluted by PE/EtOAc to obtain five subfractions (EA1-EA5). Fr.EA3-4 was subjected to repeated silica gel CC, ODS CC, Sephadex gel CC, and semipreparative HPLC to generate compound **4** (4.5 mg, *t*_R_ 13.1 min, CH_3_OH/H_2_O, 60:40 *v*/*v*), compound **9** (64.1 mg, *t*_R_ 12.3 min, CH_3_OH/H_2_O, 60:40 *v*/*v*), compound **10** (6.2 mg, *t*_R_ 13.9 min, CH_3_OH/H_2_O, 60:40 *v*/*v*), compound **11** (11.6 mg, *t*_R_ 14.3 min, CH_3_OH/H_2_O, 60:40 *v*/*v*), and compound **13** (20.3 mg, *t*_R_ 4.7 min, CH_3_OH/H_2_O, 60:40 *v*/*v*). Fr. EB (47.2 g) was subjected to silica gel CC eluted by PE/EtOAc to obtain fourteen subfractions (EB1-EB14). Compound **6** (65.3 mg, *t*_R_ 15.3 min, CH_3_OH/H_2_O, 60:40 *v*/*v*) and compound **8** (13.3 mg, *t*_R_ 8.1 min, CH_3_OH/H_2_O, 60:40 *v*/*v*) were obtained after repeated silica gel CC, ODS CC, Sephadex gel CC, and semipreparative HPLC. Fr. EC (50.50 g) was subjected to silica gel CC eluted by CH_2_Cl_2_/CH_3_OH to obtain twelve subfractions (EC1-EC12). Fr. EC-6 was eluted with 60% methanol on an ODS CC to obtain ten subfractions. Fr. EC6-5 was subjected to repeated silica gel CC, Sephadex gel CC, and semi-preparative HPLC to afford compound **5** (55.3 mg, *t*_R_ 15.3 min, CH_3_OH/H_2_O, 60:40 *v*/*v*), compound **7** (75.9 mg, *t*_R_ 13.2 min, CH_3_OH/H_2_O, 60:40 *v*/*v*), and compound **12** (7.3 mg, *t*_R_ 13.7 min, CH_3_OH/H_2_O, 60:40 *v*/*v*).

### 3.4. Preparation of Spicachlorantin E (**1**), Fortunilide E (**2**), and Chololactone B (**3**)

Purified shizukaol E, chololactone F, and chloranhololide D (50.0 mg) were resolved in a MeOH-H_2_O solution (60:40, *v*/*v*) in a heated water bath (25 °C), and the fluids were constantly replenished. The solutions were subjected to semipreparative HPLC to prepare converted compounds every two days. After accumulating for a month, chololactone B (5.5 mg), fortunilide E (2.5 mg), and spicachlorantin E (2.6 mg) were prepared.

### 3.5. HPLC-HR-MS Analysis of the Conversion Phenomena

LC−MS was performed using a Dionex Ultimate 3000 and Thermo Q Exactive plus instrument (Dionex, Sunnyvale, CA, USA). The ion source was an electric spray ionization source (ESI), with positive and negative ions scanning alternately. The scanning mode was full scan data dependency two-stage scanning (full scan/ddMS2), the scanning range was 100~1000 Da, the capillary temperature was 350 °C, the spray voltage in negative mode was 3800 V, the spray voltage in positive mode was 3200 V, the sheath gas was 35 arb, and the auxiliary gas was 15 arb. MS2 uses low, medium, and high collision energies. The positive and negative ion modes were 20 V, 40 V, and 60 V. The primary mass spectrometry resolution was FullScan 70000 FWHM (full width at half maximum), and the secondary mass spectrometry resolution was MS/MS 17500 FWHM. The data were analyzed using Xcalibur (version 4.1).

### 3.6. Theoretical Calculations

Gaussian 16 software was used for the quantum chemical calculation. B3LYP-6-311 g was selected as the base group to calculate the free energy of the reaction. The geometric optimization of the material was first performed, and then, single-point energy calculations were calculated. The method used to calculate energy is ΔG = AB free energy—A free energy—B free energy. The energy levels of the HOMO and LUMO were also calculated using b3lyp-6-311g.

### 3.7. Cell Culture

RAW 264.7 mouse macrophages were obtained from Dr. Chunhua Ma of Nanjing University of Chinese Medicine and were cultured in DMEM with 3.0 mM glutamine, 100 U/mL penicillin, 100 U/mL streptomycin, and 10% heat-inactivated fetal bovine serum at 37 °C in a humidified incubator with 5% CO_2_. In all experiments, macrophages were incubated in the presence or absence of different concentrations of **1** (2.5, 5.0, and 10.0 μM), which were solubilized with DMSO and added 1 h before LPS (1.0 μg/mL) stimulation.

### 3.8. Measurement of Nitrite Concentration

Raw 264.7 macrophages were cultured at a density of 2 × 10^4^ cells/mL in 96-well plates. Then, they were incubated with or without LPS (1.0 g/mL) in the absence or presence of different concentrations of **1** (2.5, 5.0, and 10.0 μM) for up to 24 h. Dexamethasone (1.0 μM) was used as a positive control. Each 50 μL of culture supernatant was mixed with an equal volume of 0.1% naphthylethylene diamine and 1.0% sulfanilamide in 2.5% phophoric acid solution (Griess reagent) and incubated at room temperature for 10 min. The concentrations of NO were determined by a sodium nitrite standard curve after measurement with an automated microplate reader.

### 3.9. Proinflammatory Cytokine (IL-1β) Assay

Compound **1** solubilized with DMSO was diluted with DMEM before treatment. RAW 264.7 cells were plated onto 24-well plates (4 × 10^5^ cells/well) and were incubated in the presence of either 1.0 mg/mL LPS alone or LPS plus various concentrations of **1** (2.5, 5.0, or 10.0 μM) for 18 h. Following the manufacturer’s protocol, a mouse ELISA kit (ebioscience, San Diego, CA, USA) was employed to analyze cell-free supernatants in the proinflammatory cytokine assays.

### 3.10. Statistical Analysis

All data are expressed as the means ± SD. The test was used to analyze the differences between sets of data with GraphPad Prism 9 software. All experiments were repeated at least three times.

## Data Availability

Data are contained within the article and supplementary materials.

## Supplementary Materials

The following supporting information can be downloaded at: https://www.mdpi.com/article/10.3390/molecules29040909/s1.

## References

[1] Yang P.Y., Jia Q., Song S.J., Huang X.X. (2023). [2+2]-Cycloaddition-derived cyclobutane natural products: Structural diversity, sources, bioactivities, and biomimetic syntheses. Nat. Prod. Rep..

[2] Yang S.P., Gao Z.B., Wang F.D., Liao S.G., Chen H.D., Zhang C.R., Hu G.Y., Yue J.M. (2007). Chlorahololides A and B, two potent and selective blockers of the potassium channel isolated from *Chloranthus holostegius*. Org. Lett..

[3] Liu Y.Y., Li Y.Z., Huang S.Q., Zhang H.W., Deng C., Song X.M., Zhang D.D., Wang W. (2022). Genus Chloranthus: A comprehensive review of its phytochemistry, pharmacology, and uses. Arab. J. Chem..

[4] Zhang D.Y., Wang X.X., Wang Y.N., Wang M., Zhuang P.Y., Jin Y., Liu H. (2021). Nine sesquiterpenoid dimers with four unprecedented types of carbon skeleton from *Chloranthus henryi* var. hupehensis. Org. Chem. Front..

[5] Wang S.Y., Sun Y.P., Li Y.Q., Xu W.J., Li Q.Q., Mu Y.B., Kong L.Y., Luo J. (2023). Rearranged Lindenane Sesquiterpenoid Trimers from *Chloranthus fortunei*: Target Discovery and Biomimetic Conversion. J. Org. Chem..

[6] Li J.X., Cui Z.R., Li Y.Y., Han C.H., Zhang Y.Q., Tang P.F., Cui L.T., Zhang H., Luo J., Kong L.Y. (2022). Chlospicenes A and B, cyclopropane cracked lindenane sesquiterpenoid dimers with anti-nonalcoholic steatohepatitis activity from *Chloranthus henryi*. Chin. Chem. Lett..

[7] Maslovskaya L.A., Savchenko A.I., Krenske E.H., Pierce C.J., Gordon V.A., Reddell P.W., Parsons P.G., Williams C.M. (2014). EBC-219: A New Diterpene Skeleton, Crotinsulidane, from the Australian Rainforest Containing a Bridgehead Double Bond. Angew. Chem. Int. Edit..

[8] Agatsuma T., Akama T., Nara S., Matsumiya S., Nakai R., Ogawa H., Otaki S., Ikeda S., Saitoh Y., Kanda Y. (2002). UCS1025A and B, new antitumor antibiotics from the fungus Acremonium species. Org. Lett..

[9] Cuesta-Rubio O., Padron A., Castro H.V., Pizza C., Rastrelli L. (2001). Aristophenones A and B. A new tautomeric pair of polyisoprenylated benzophenones from Garcinia aristata. J. Nat. Prod..

[10] Liang X., Huang Z.H., Ma X., Qi S.H. (2018). Unstable Tetramic Acid Derivatives from the Deep-Sea-Derived Fungus Cladosporium sphaerospermum EIODSF 008. Mar. Drugs.

[11] Sun Y.W., Liu G.M., Huang H., Yu P.Z. (2012). Chromone derivatives from Halenia elliptica and their anti-HBV activities. Phytochemistry.

[12] Xu L., Guo F.W., Zhang X.Q., Zhou T.Y., Wang C.J., Wei M.Y., Gu Y.C., Wang C.Y., Shao C.L. (2022). Discovery, total syntheses and potent anti-inflammatory activity of pyrrolinone-fused benzoazepine alkaloids Asperazepanones A and B from Aspergillus candidus. Commun. Chem..

[13] Zhao P., Huang X.X., Song S.J. (2022). The hypothesis of tautomeric equilibrium between epimers in ciquitins A and B. J. Asian Nat. Prod. Res..

[14] Zhang J., Zhang Q.Y., Tu P.F., Xu F.C., Liang H. (2018). Mucroniferanines A–G, Isoquinoline Alkaloids from *Corydalis mucronifera*. J. Nat. Prod..

[15] Cao C.M., Zhang H.P., Gallagher R.J., Timmermann B.N. (2013). Withanolide artifacts formed in methanol. J. Nat. Prod..

[16] Wu J.P., Li L.Y., Li J.R., Yu M., Zhao J.P., Xu Q.M., Gu Y.C., Zhang T., Zou Z.M. (2022). Silencing Tautomerization to Isolate Unstable Physalins from *Physalis minima*. J. Nat. Prod..

[17] Kawabata J., Mizutani J. (1992). Dimeric sesquiterpenoid esters from *Chloranthus serratus*. Phytochemistry.

[18] Yang S.P., Gao Z.B., Wu Y., Hu G.Y., Yue J.M. (2008). Chlorahololides C–F: A new class of potent and selective potassium channel blockers from *Chloranthus holostegius*. Tetrahedron.

[19] Shen C.P., Luo J.G., Yang M.H., Kong L.Y. (2017). Sesquiterpene dimers from the roots of *Chloranthus holostegius* with moderate anti-inflammatory activity. Phytochemistry.

[20] Kawabata J., Fukushi E., Mizutani J. (1995). Sesquiterpene Dimers from *Chloranthus japonicus*. Phytochemistry.

[21] Bian X.X., Zhao X., Liu S.S., Wu L., Yin X.W., Shen C.P. (2022). Sesquiterpene dimers from *Chloranthus fortunei* and their protection activity against acute lung injury. Fitoterapia.

[22] Zhou B., Wu Y., Dalal S., Merino E.F., Liu Q.F., Xu C.H., Yuan T., Ding J., Kingston D.G.I., Cassera M.B. (2017). Nanomolar Antimalarial Agents against Chloroquine-Resistant Plasmodium falciparum from Medicinal Plants and Their Structure-Activity Relationships. J. Nat. Prod..

[23] Wu J.L., Lu Y.S., Tang B.C., Peng X.S. (2018). Total syntheses of shizukaols A and E. Nat. Commun..

[24] Kim S.Y., Kashiwada Y., Kawazoe K., Murakami K., Sun H.D., Li S.L., Takaishi Y. (2009). Spicachlorantins C-F, hydroperoxy dimeric sesquiterpenes from the roots of *Chloranthus spicatus*. Tetrahedron Lett..

[25] Kong L.Y., Tan R.X. (2015). Artemisinin, a miracle of traditional Chinese medicine. Nat. Prod. Rep..

[26] Gordon S., Martinez F.O. (2010). Alternative Activation of Macrophages: Mechanism and Functions. Immunity.

[27] Ren Y., Ma Y.S., Zhang Z.D., Qiu L.Y., Zhai H.H., Gu R.M., Xie Y.P. (2019). Total Alkaloids from Bamboo Shoots and Bamboo Shoot Shells of Pleioblastus amarus (Keng) Keng f. and Their Anti-Inflammatory Activities. Molecules.

[28] Jiang F., Zang L.H., Miao X.Q., Jia F., Wang J., Zhu M.L., Gong P., Jiang N., Zhai X. (2019). Design, synthesis and anti-inflammatory evaluation of novel pyrrolo [2,3-] pyrimidin derivatives as potent JAK inhibitors. Bioorgan. Med. Chem..

[29] Kim D., Chun S.H., Oh N.S., Lee J.Y., Lee K.W. (2019). Anti-inflammatory activities of Maillard reaction products from whey protein isolate fermented by 4M13 in lipopolysaccharide- stimulated RAW264.7 cells. J. Dairy Sci..

[30] Banskota S., Wang H.Q., Kwon Y.H., Gautam J., Gurung P., Haq S., Hassan F.M.N., Bowdish D.M., Kim J.A., Carling D. (2021). Salicylates Ameliorate Intestinal Inflammation by Activating Macrophage AMPK. Inflamm. Bowel. Dis..

[31] Ferrer M.D., Busquets-Cortés C., Capó X., Tejada S., Tur J.A., Pons A., Sureda A. (2019). Cyclooxygenase-2 Inhibitors as a Therapeutic Target in Inflammatory Diseases. Curr. Med. Chem..

[32] Shen C.P., Luo J.G., Yang M.H., Kong L.Y. (2015). Cafestol-Type Diterpenoids from the Twigs of Tricalysia fruticosa with Potential Anti-inflammatory Activity. J. Nat. Prod..

[33] Yang B.Y., Kong L.Y., Wang X.B., Zhang Y.M., Li R.J., Yang M.H., Luo J.G. (2016). Nitric Oxide Inhibitory Activity and Absolute Configurations of Arylalkenyl α, β-Unsaturated δ/γ-Lactones from Cryptocarya concinna. J. Nat. Prod..

[34] Li L.N., Zhou M.M., Xue G.M., Wang W.L., Zhou X.W., Wang X.B., Kong L.Y., Luo J.G. (2018). Bioactive seco-abietane rearranged diterpenoids from the aerial parts of Salvia prionitis. Bioorg. Chem..

