# Supplementary material for: Anti-Inflammatory Peroxidized Chlorahololide-Type Dimers Are Artifacts of Shizukaol-Type Dimers: From Phenomena Discovery and Confirmation to Potential Underlying Mechanism
Source: Molecules. 2024 Feb 19;29(4):909. doi: 10.3390/molecules29040909 (PMC10893383; doi:10.3390/molecules29040909)
Supplement: Supplementary file 1 [file molecules-29-00909-s001.zip › molecules-2831556-supplementary.pdf]

Supporting information

Peroxidized Chloraholide-Type Dimers Are Artifacts of Shizukaol-Type Dimers: from Phenomena Discovery and Confirmation to Potential Underlying Mechanism

Xiu-wen Yin[1#], Lan Wu[1#], Meng-yu Yang[1], Yan-fang Chen[1], Ming Zhang[1], Fu-cai, Ren[1], Fu-rong Yang[1], Xiang-dong Pu[1], Bing-yuan Yang*[3], Zhi-Jun Zhang[*2], Chuan-pu Shen[*1]

[1]Anhui Provincial laboratory of inflammatory and immunity disease, Anhui Institute of Innovative Drugs, School of pharmacy, Anhui Medical University, Hefei 230032, People's Republic of China

[2]School of Pharmacy, Hubei University of Science and Technology, Xianning, 437100, China

[3]Guangxi Key Laboratory of Plant Functional Phytochemicals and Sustainable Utilization, Guangxi Institute of Botany, Guangxi Zhuang Autonomous Region and Chinese Academy of Sciences, Guilin 541006, People's Republic of China

[#] They contribute equally to this work.

*To whom correspondence should be addressed.

E-mail: 2016500001@ ahmu.edu.cn (Shen, C.P.); zzj@hbust.edu.cn (Zhang, Z. J.); yby@gxib.cn (Yang, B.Y.)

The list of contents	pages

Part I

Figure S1. The result of the UPLC-HR-MS analysis of positive and negative ion flow. 3

Part II

Figure S2. The compounds isolated from the roots of *Chloranthus fortunei.* 4
Figure S3. The confirmation of the conversion of compound **6**. 5
Figure S4. The confirmation of the conversion of compound **9**. 6
Figure S5. The confirmation of the conversion of compound **10**. 7
Figure S6. The confirmation of the conversion of compound **11**. 8
Figure S7. The confirmation of the conversion of compound **12**. 9
Figure S8. The ^{1}H NMR spectrum of compound **1** ($CDCl_3$, 500MHz) 10
Figure S9. The ^{13}C NMR spectrum of compound **1** ($CDCl_3$, 125MHz) 10
Figure S10. The ^{1}H NMR spectrum of compound **2** ($CDCl_3$, 500MHz) 11
Figure S11. The ^{13}C NMR spectrum of compound **2** ($CDCl_3$, 125MHz) 11
Figure S12. The HSQC spectrum of compound **2** in $CDCl_3$ 12
Figure S13. The HMBC spectrum of compound **2** in $CDCl_3$ 12
Figure S14. The ROESY spectrum of compound **2** in $CDCl_3$ 13
Figure S15. The ^{1}H NMR spectrum of compound **3** ($CDCl_3$, 500MHz) 13
Figure S16. The ^{13}C NMR spectrum of compound **3** ($CDCl_3$, 125MHz) 14
Figure S17. The HSQC spectrum of compound **3** in $CDCl_3$ 14
Figure S18. The HMBC spectrum of compound **3** in $CDCl_3$ 15
Figure S19. The ROESY spectrum of compound **3** in $CDCl_3$ 15
Figure S20. The ^{1}H NMR spectrum of compound **4** ($CDCl_3$, 500MHz) 16
Figure S21. The ^{13}C NMR spectrum of compound **4** ($CDCl_3$, 125MHz) 16
Figure S22. The ^{1}H NMR spectrum of compound **5** ($CDCl_3$, 500MHz) 17
Figure S23. The ^{13}C NMR spectrum of compound **5** ($CDCl_3$, 125MHz) 17
Figure S24. The ^{1}H NMR spectrum of compound **6** ($CDCl_3$, 500MHz) 18
Figure S25. The ^{13}C NMR spectrum of compound **6** ($CDCl_3$, 125MHz) 18
Figure S26. The ^{1}H NMR spectrum of compound **7** ($CDCl_3$, 500MHz) 19
Figure S27. The ^{13}C NMR spectrum of compound **7** ($CDCl_3$, 125MHz) 19
Figure S28. The ^{1}H NMR spectrum of compound **8** ($CDCl_3$, 500MHz) 20
Figure S29. The ^{13}C NMR spectrum of compound **8** ($CDCl_3$, 125MHz) 20
Figure S30. The ^{1}H NMR spectrum of compound **9** ($CDCl_3$, 500MHz) 21
Figure S31. The ^{13}C NMR spectrum of compound **9** ($CDCl_3$, 125MHz) 21
Figure S32. The ^{1}H NMR spectrum of compound **10** ($CDCl_3$, 500MHz) 22
Figure S33. The ^{13}C NMR spectrum of compound **10** ($CDCl_3$, 125MHz) 22
Figure S34. The ^{1}H NMR spectrum of compound **11** ($CDCl_3$, 500MHz) 23
Figure S35. The ^{13}C NMR spectrum of compound **11** ($CDCl_3$, 125MHz) 23
Figure S36. The ^{1}H NMR spectrum of compound **12** ($CDCl_3$, 500MHz) 24
Figure S37. The ^{13}C NMR spectrum of compound **12** ($CDCl_3$, 125MHz) 24
Figure S38. The ^{1}H NMR spectrum of compound **13** ($CDCl_3$, 500MHz) 25
Figure S39. The ^{13}C NMR spectrum of compound **13** ($CDCl_3$, 125MHz) 25
Figure S40. HPLC chromatography of **8** after one month of the purification. 26
Figure S41. HPLC chromatography of **11** after one month of the purification. 26
S1. ^{1}H and ^{13}C NMR Spectroscopic Data for Compounds **1**. 27
S2. ^{1}H and ^{13}C NMR Spectroscopic Data for Compounds **2**. 28
S3. ^{1}H and ^{13}C NMR Spectroscopic Data for Compounds **3**. 29
S4. The ECD spectrum of compounds **1-6** 30-32
S5-11. The DFT calculation parameter of article 33-45

Part I Complementary experiment confirming that compounds 1, 2 and 3 are artifacts generated during purification and storage process.

The LC-MS experiment was undertaken to confirm whether compound **1**, **2** and **3** were existed naturally in the crude extract. The roots of *Chloranthus fortunei* (50.0 g) were ultrasonically extracted with 300 mL of EtOH for 2 h at room temperature. The extract was concentrated by rotary evaporation at 45℃ under reduced pressure, and redissolved sequentially with MeOH (5 mL) which was analyzed by HPLC-DAD/ESI-HR-MS used the MeOH/H_2O (30:70-90:10, 0-60min; 90:10-90:10, 60-70min) at 0.8mL/min as eluents to obtained the detailed information. As shown in figure 1, we do not detect compounds **1**, **2**, and **3** in the extraction of *Chloranthus fortunei,* suggesting that **1**, **2**, and **3** were artifacts generated from the purification procedure.

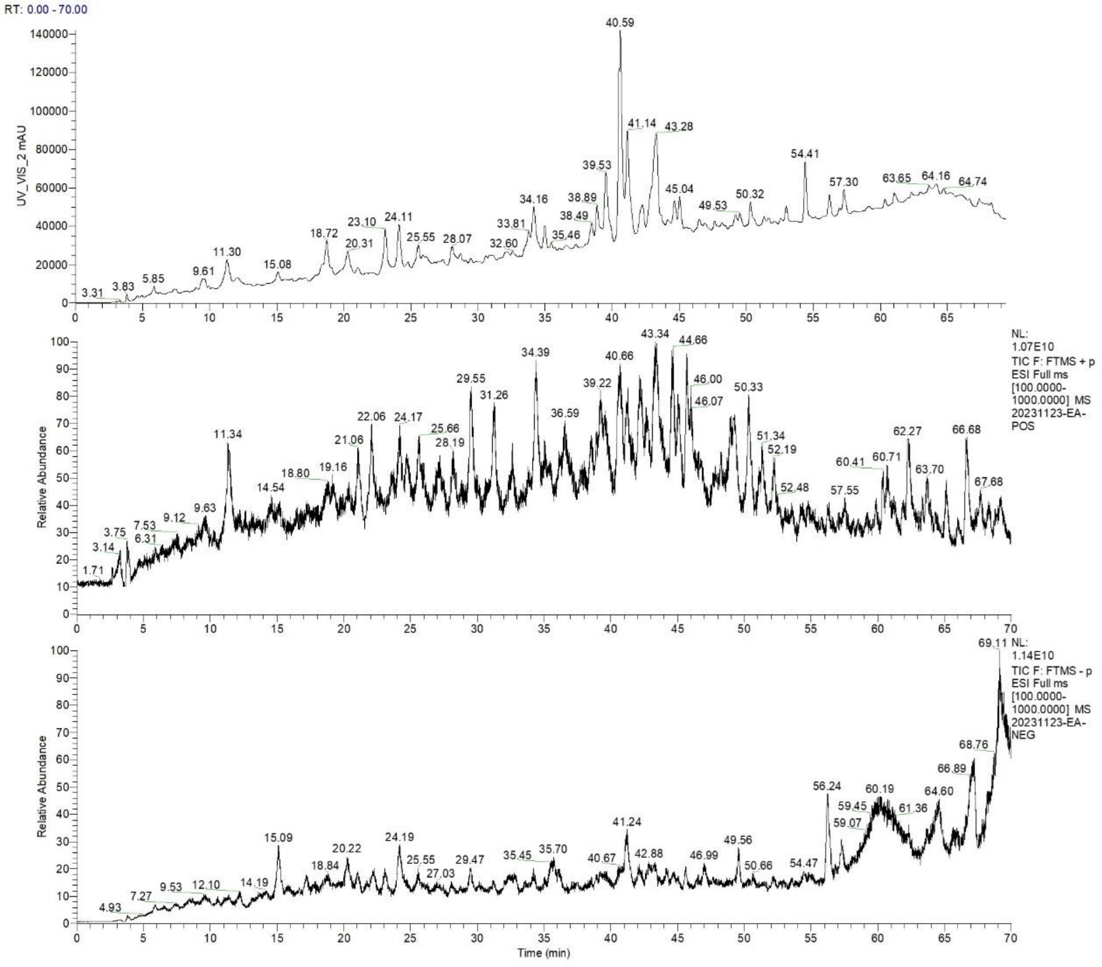

Figure S1. The result of the UPLC-HR-MS analysis of positive and negative ion flow.

Part II Figures

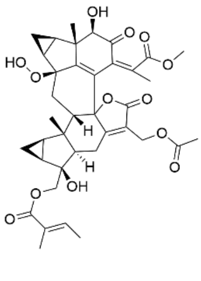

1

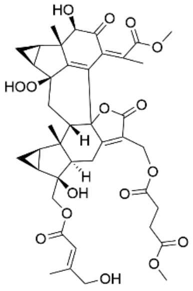

2

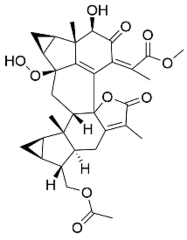

3

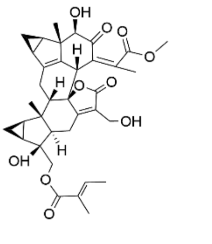

4

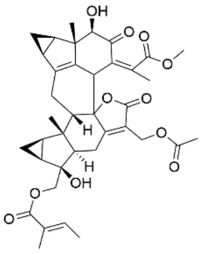

5

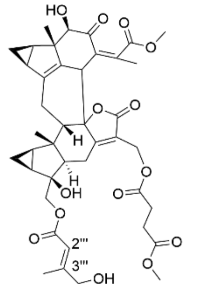

6 2''',3'''-E

12 2''',3'''-Z

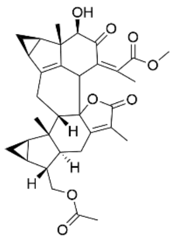

7

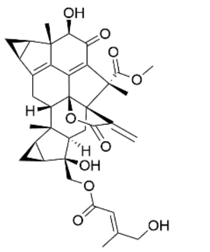

8

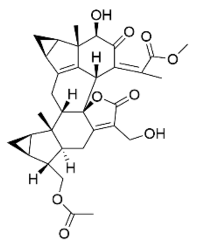

9

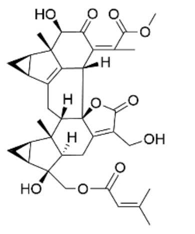

10

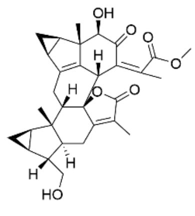

11

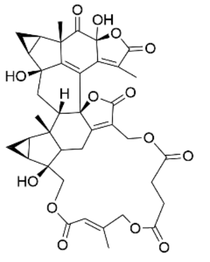

13

Figure S2. The compounds isolated from the roots of *Chloranthus fortunei.*

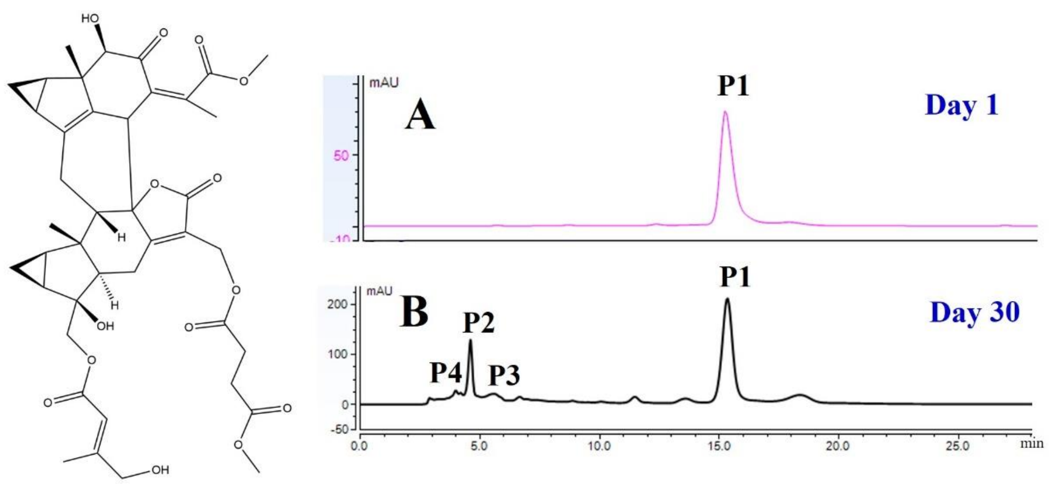

cholo lactone F(6)

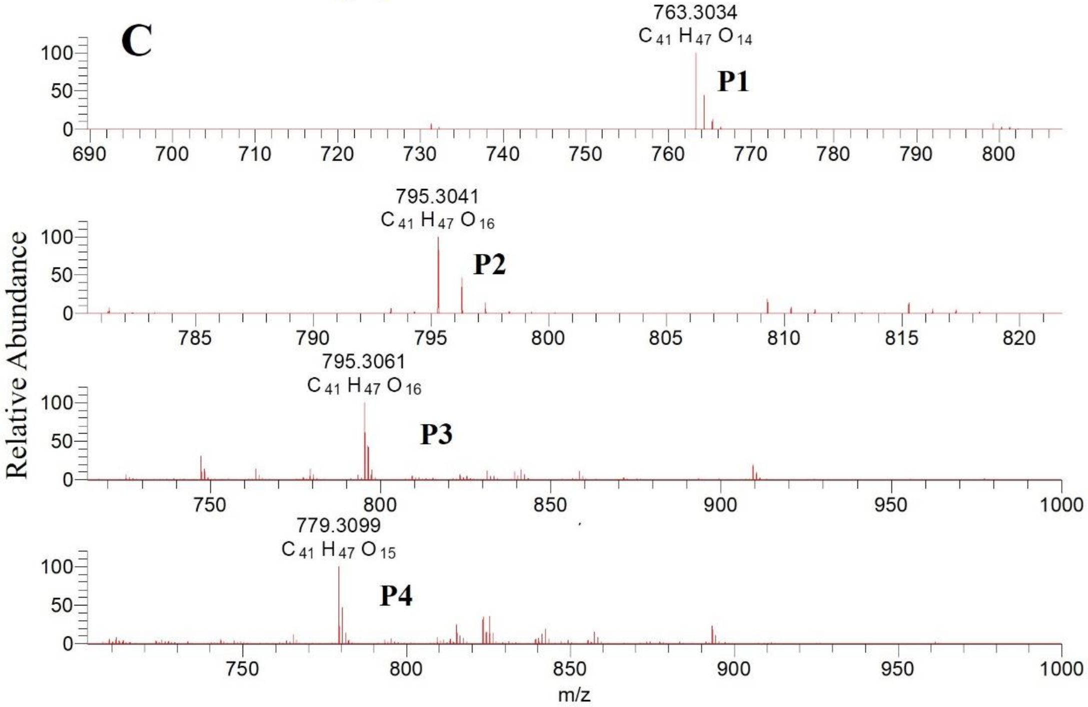

Figure S3. The confirmation of the conversion of compound **6. A)** HPLC chromatography of **6** after one day of the purification. **B)** HPLC chromatography of **6** after one month of the purification. **C)** The result of the UPLC-HR-MS analysis of **6** after one month of the purification.

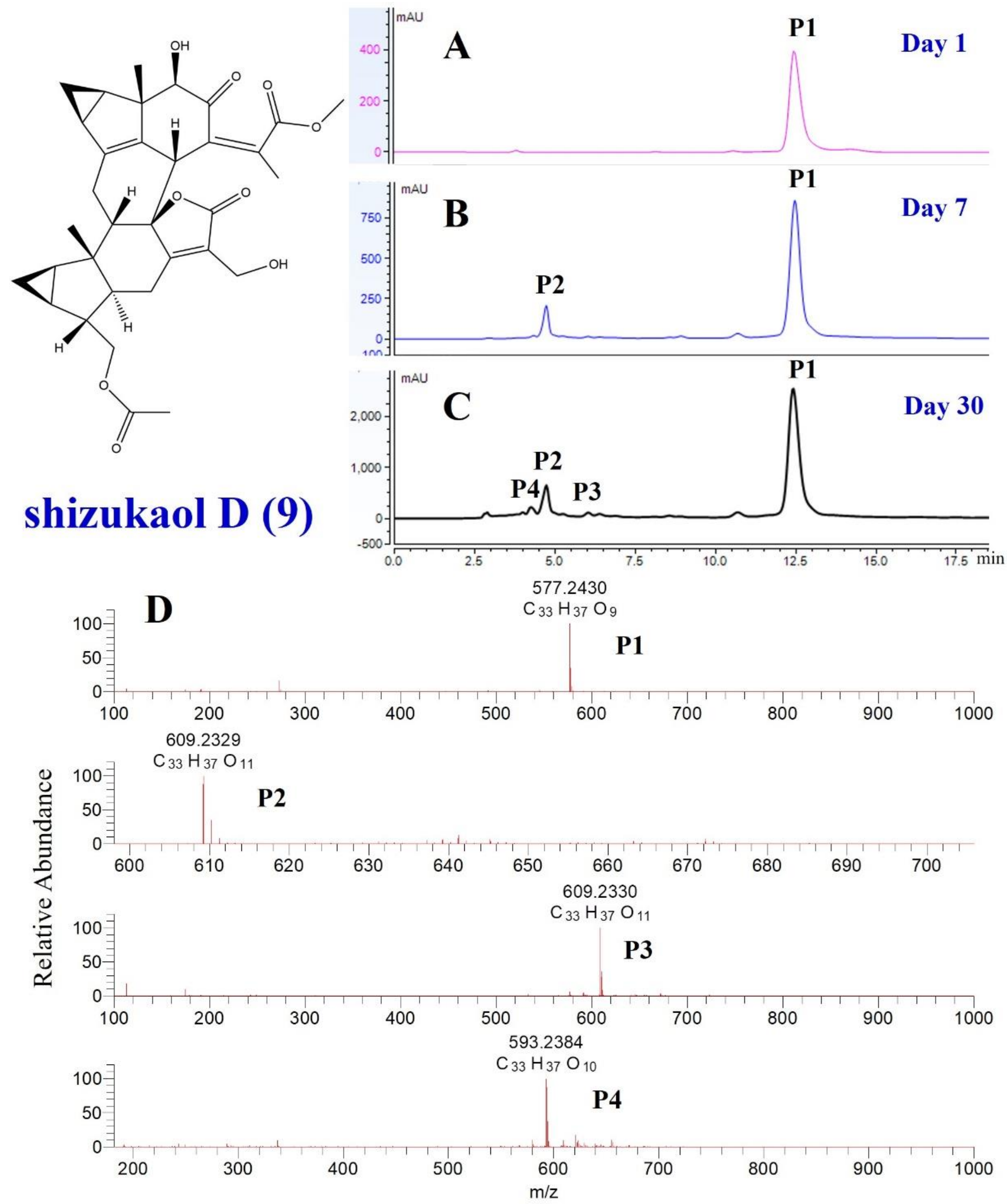

Figure S4. The confirmation of the conversion of compound **9. A)** HPLC chromatography of **9** after one day of the purification. **B)** HPLC chromatography of **9** after one week of the purification. **C)** HPLC chromatography of **9** after one month of the purification. **D)** The result of the UPLC-HR-MS analysis of **9** after one month of the purification.

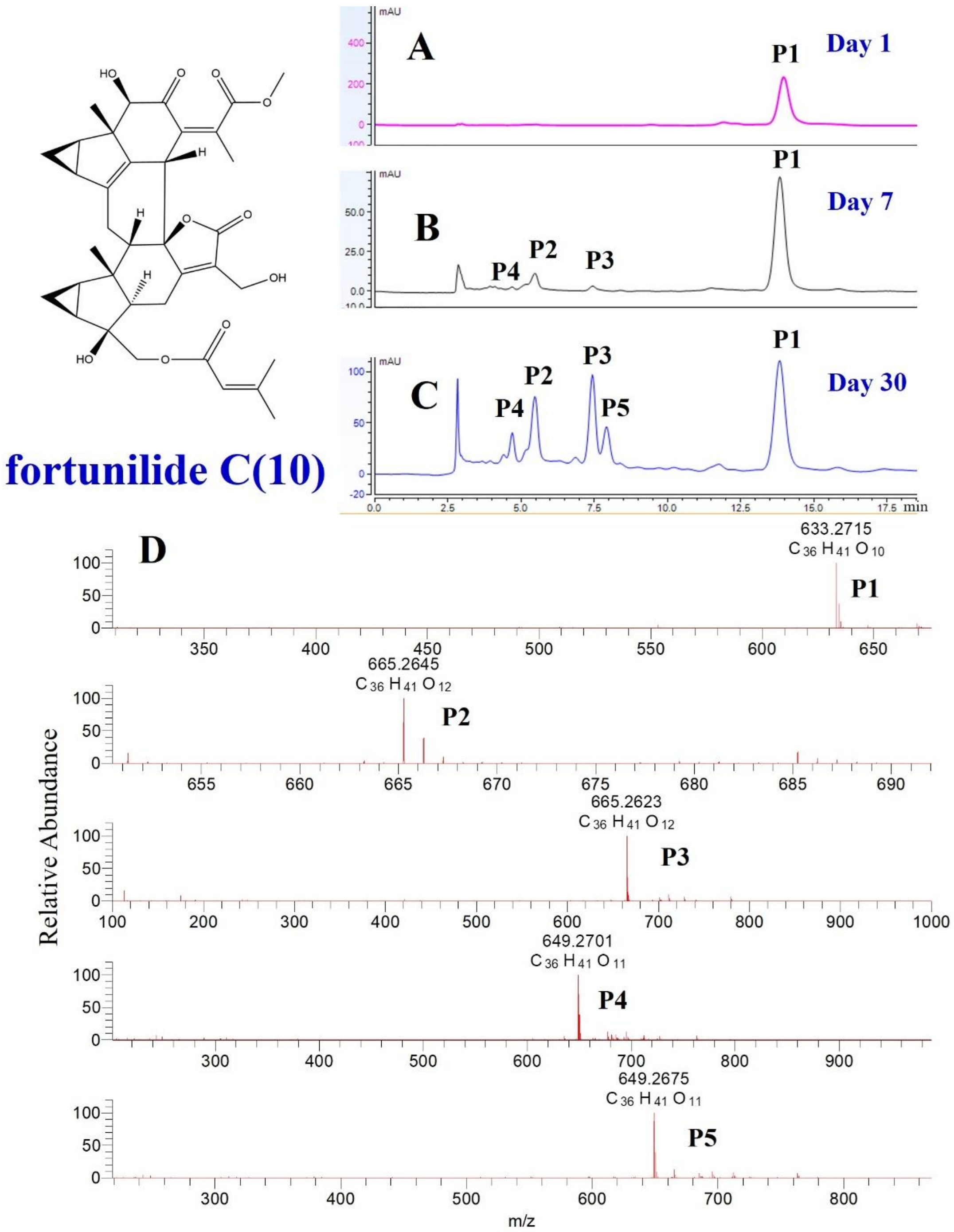

Figure S5. The confirmation of the conversion of compound **10.** **A)** HPLC chromatography of **10** after one day of the purification. **B)** HPLC chromatography of **10** after one week of the purification. **C)** HPLC chromatography of **10** after one month of the purification. **D)** The result of the UPLC-HR-MS analysis of **10** after one month of the purification.

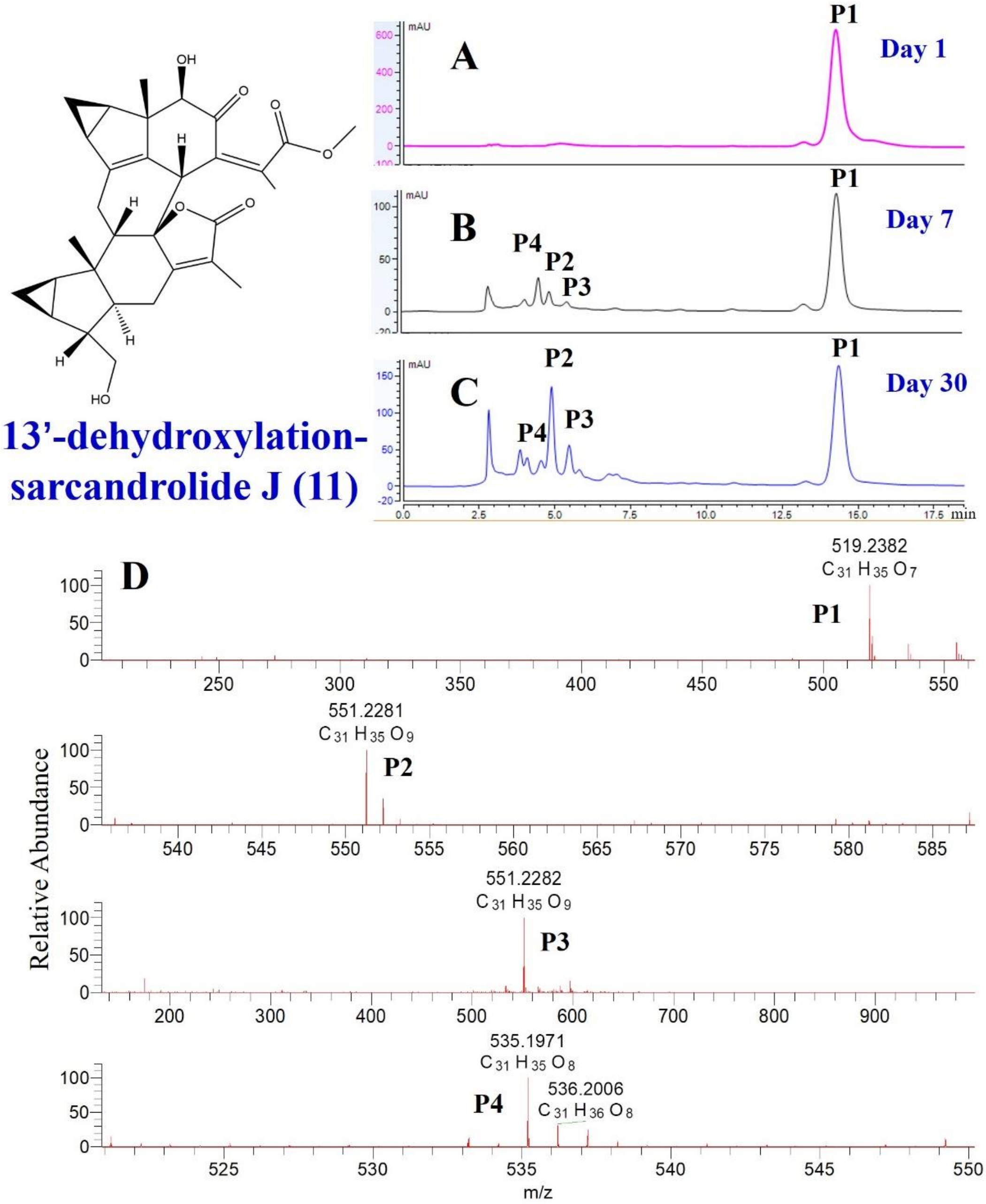

Figure S6. The confirmation of the conversion of compound **11. A)** HPLC chromatography of **11** after one day of the purification. **B)** HPLC chromatography of **11** after one week of the purification. **C)** HPLC chromatography of **11** after one month of the purification. **D)** The result of the UPLC-HR-MS analysis of **11** after one month of the purification.

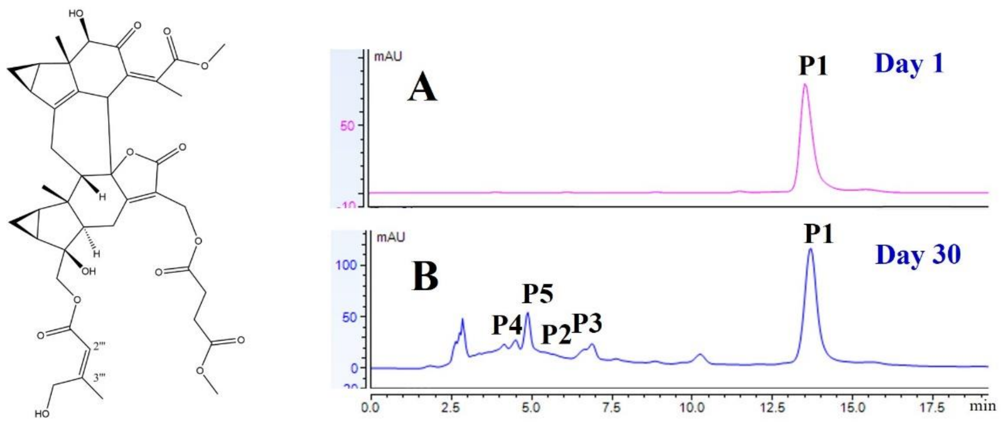

sarglabolide J(12)

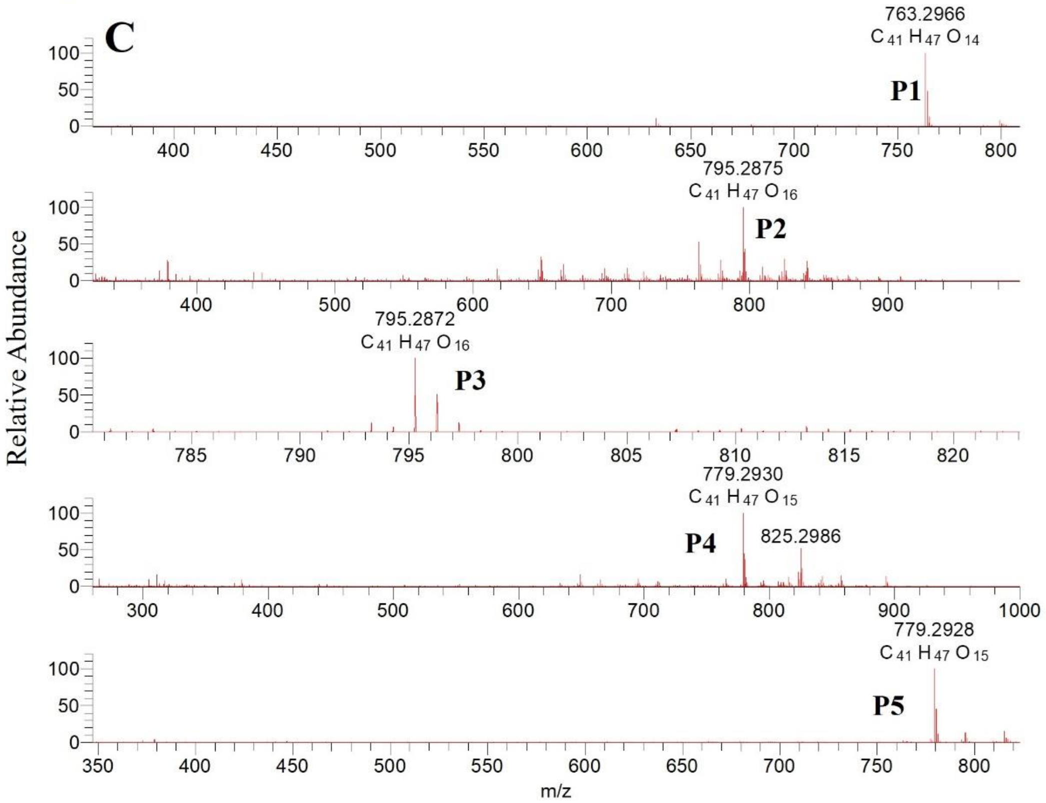

Figure S7. The confirmation of the conversion of compound **12. A)** HPLC chromatography of **12** after one day of the purification. **B)** HPLC chromatography of **12** after one month of the purification. **C)** The result of the UPLC-HR-MS analysis of **12** after one month of the purification.

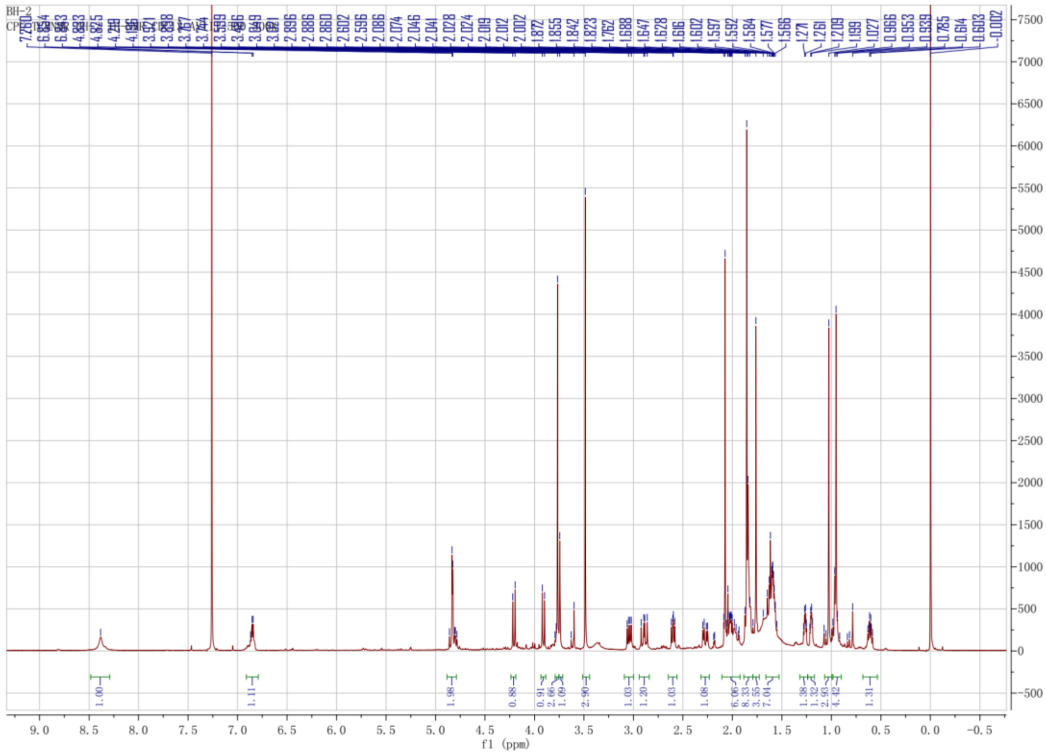

Figure S8. The ^{1}H NMR spectrum of compound **1** ($CDCl_3$, 500MHz)

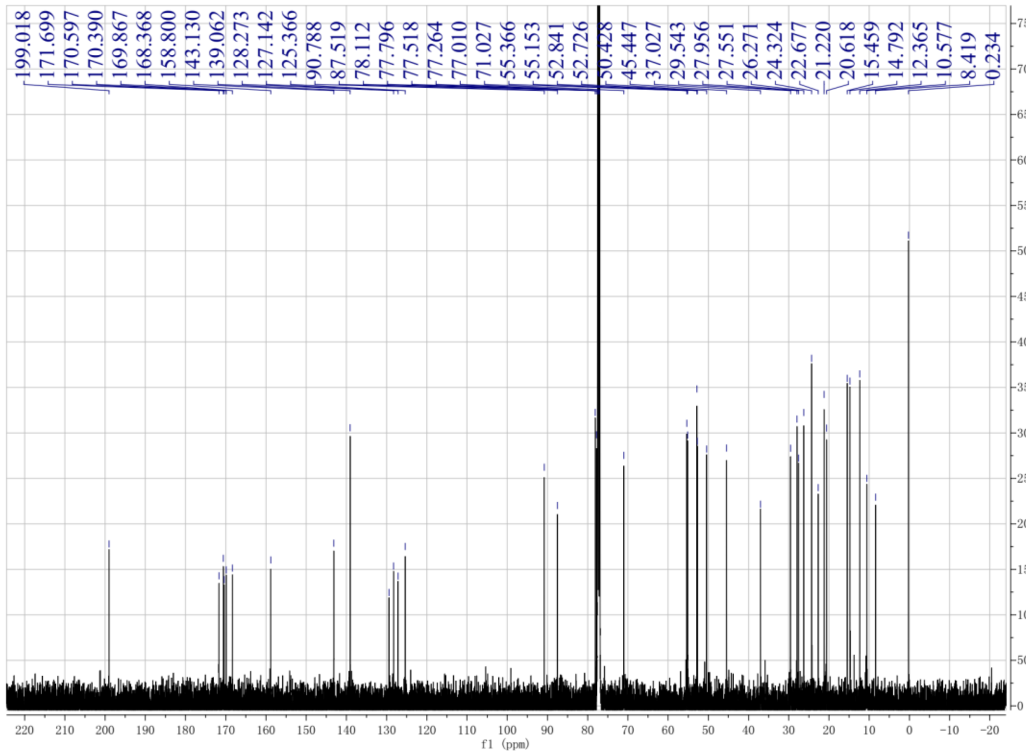

Figure S9. The ^{13}C NMR spectrum of compound **1** ($CDCl_3$, 125MHz)

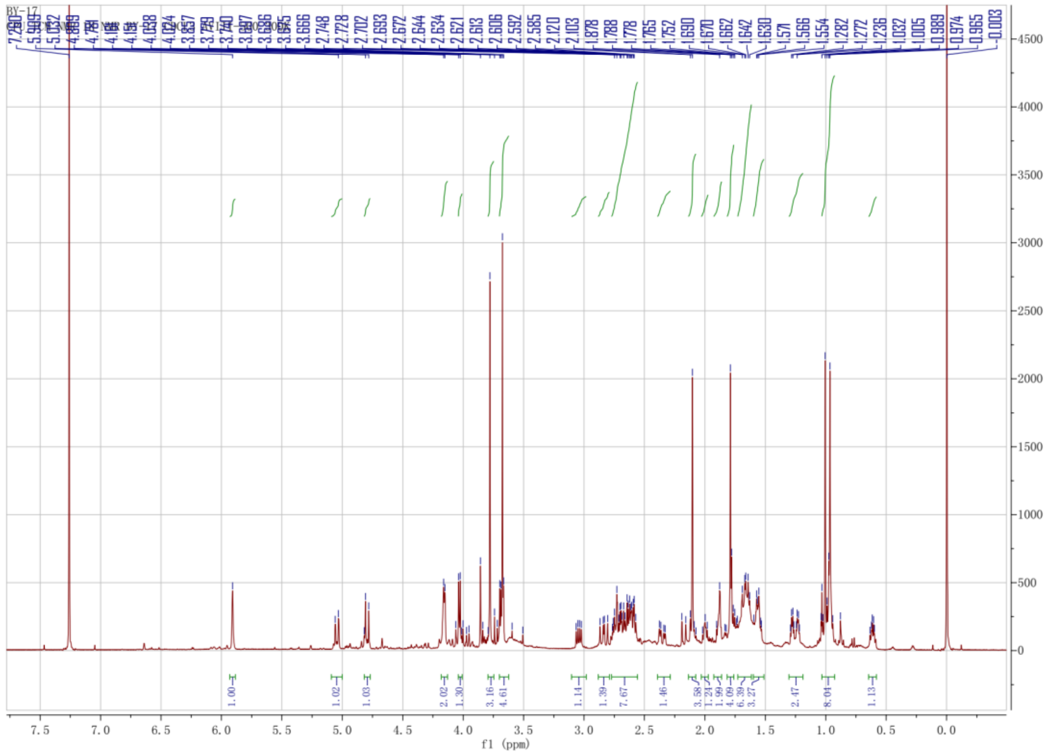

Figure S10. The ^{1}H NMR spectrum of compound **2** ($CDCl_3$, 500MHz)

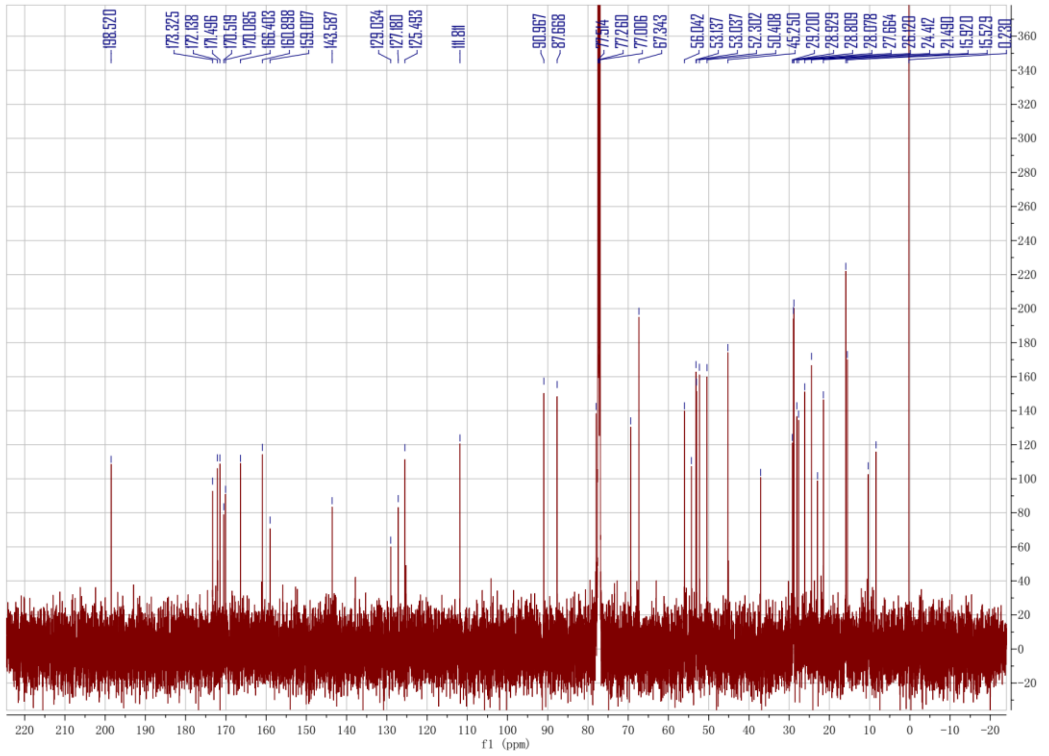

Figure S11. The ^{13}C NMR spectrum of compound **2** ($CDCl_3$, 125MHz)

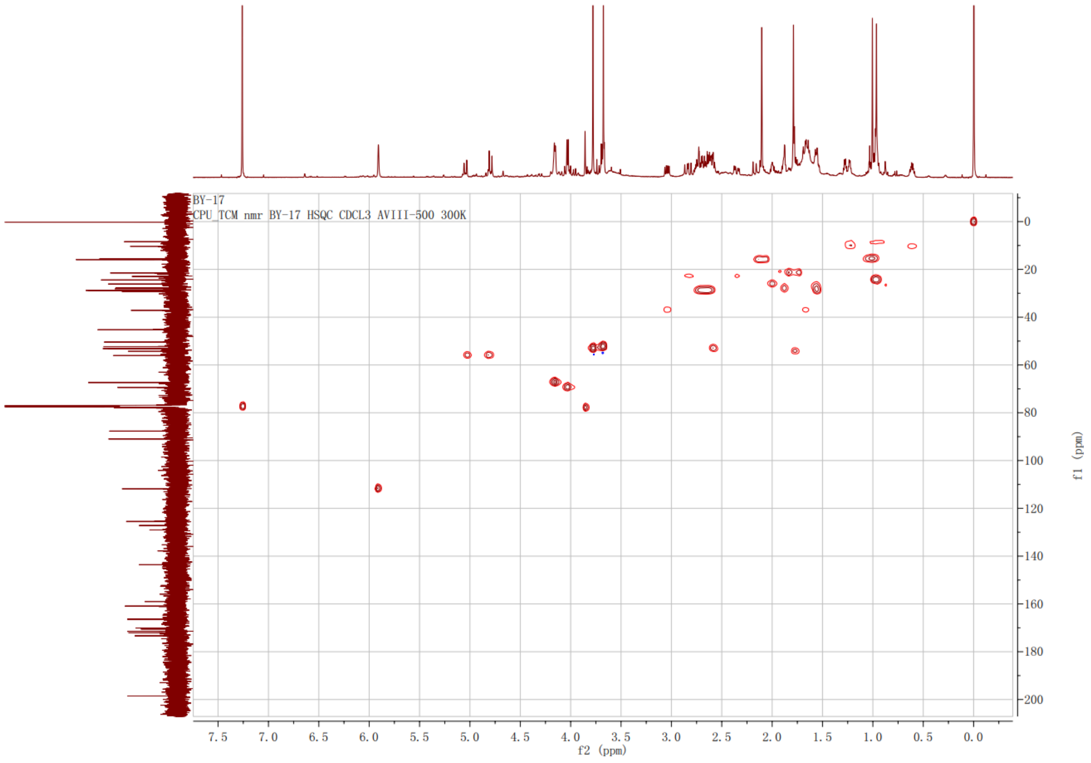

Figure S12. The HSQC spectrum of compound **2** in $CDCl_3$

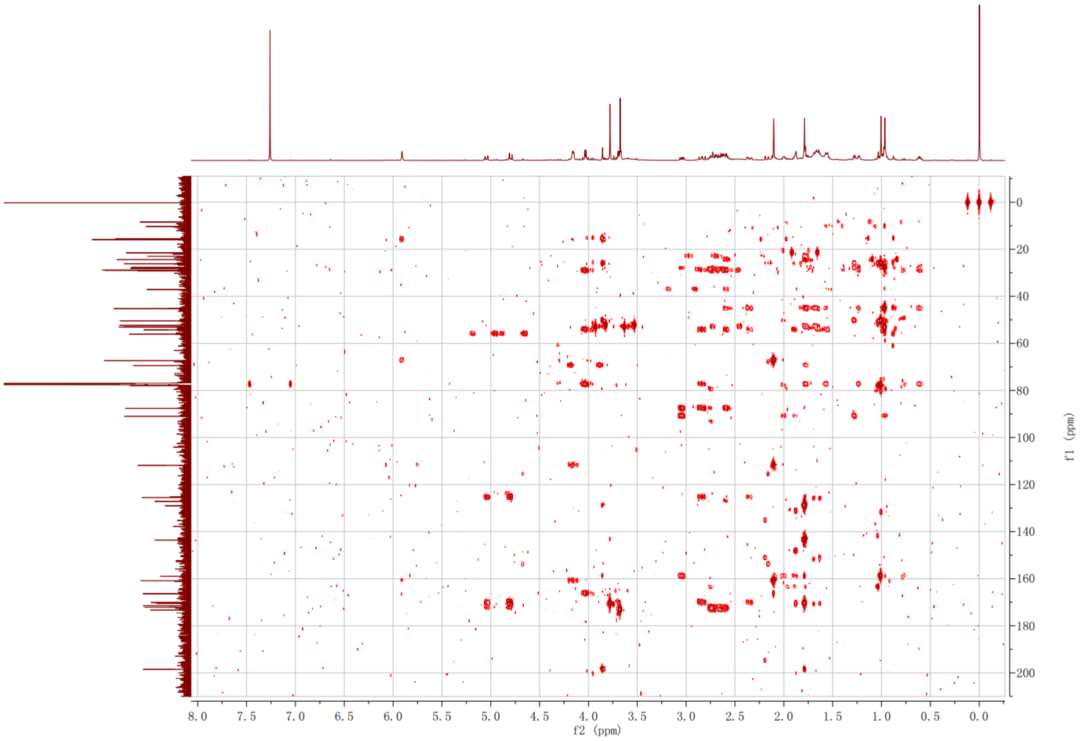

Figure S13. The HMBC spectrum of compound **2** in $CDCl_3$

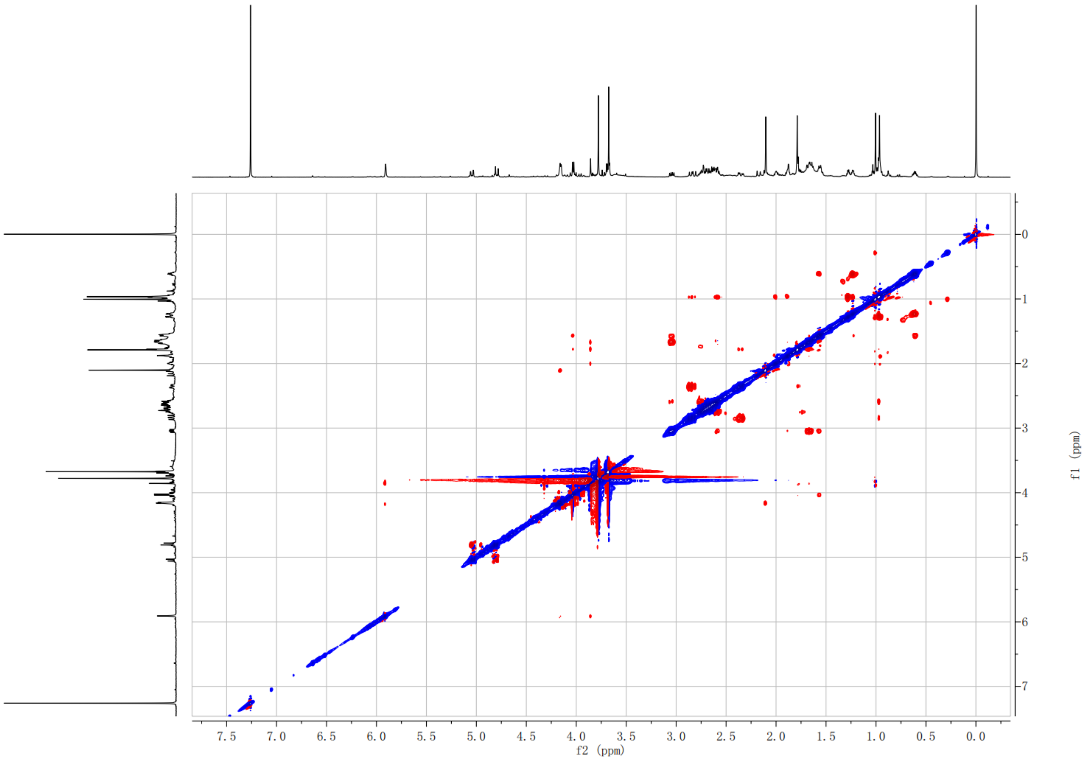

Figure S14. The ROESY spectrum of compound **2** in $CDCl_3$

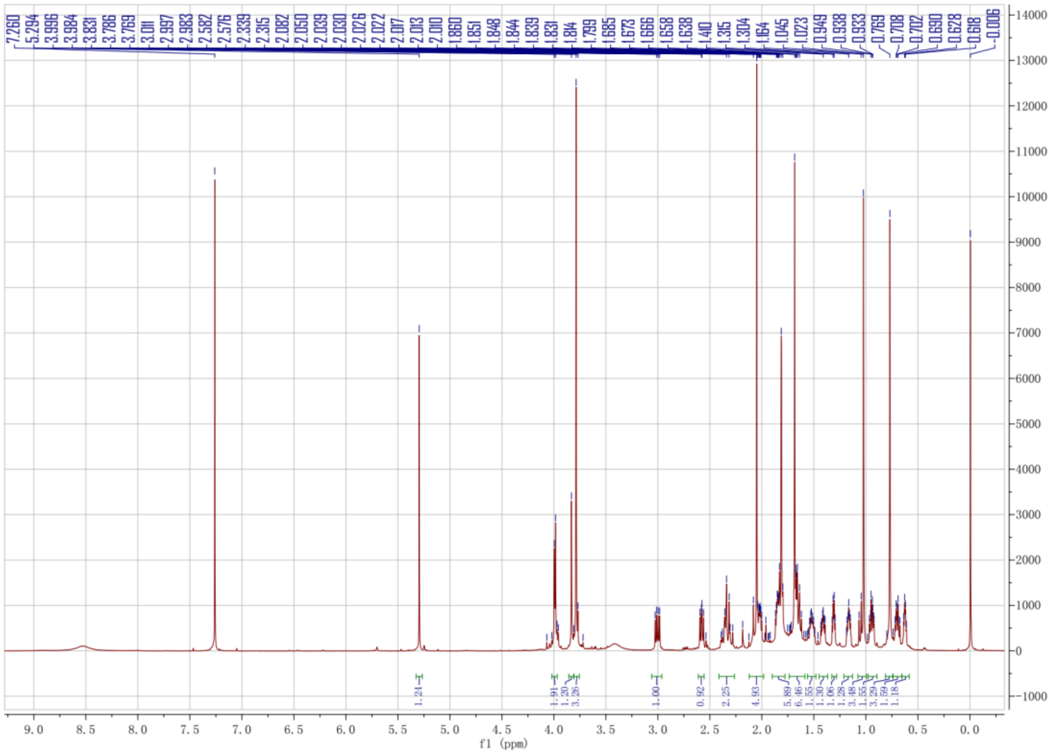

Figure S15. The ^{1}H NMR spectrum of compound **3** ($CDCl_3$, 500MHz)

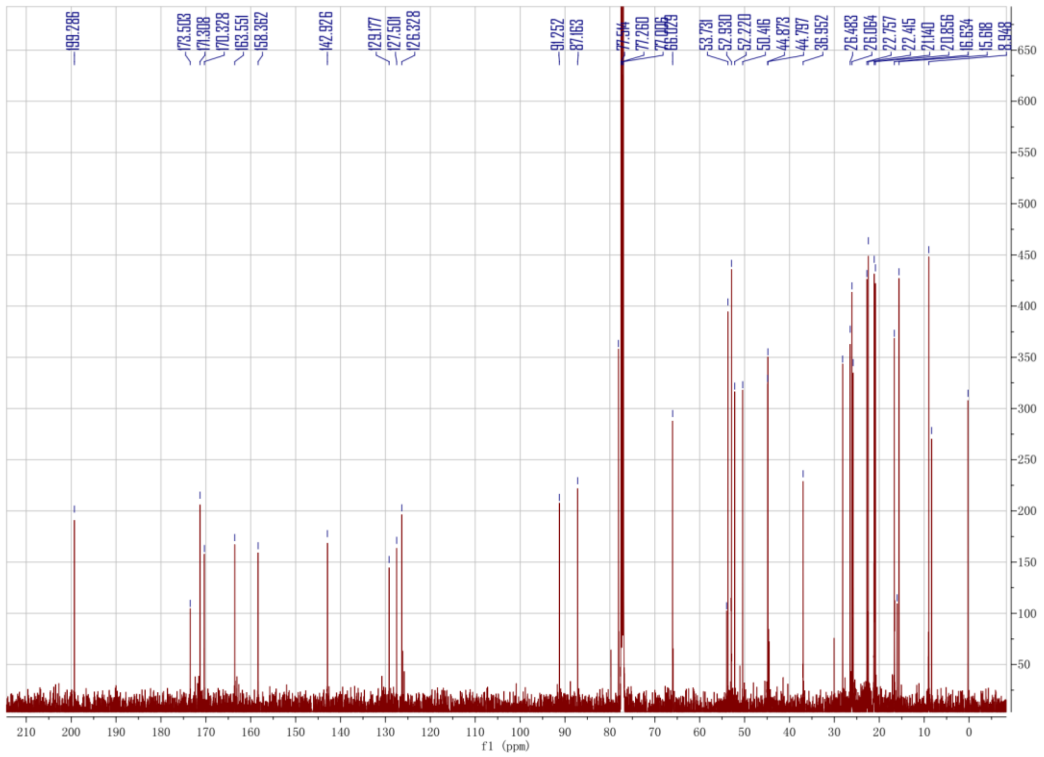

Figure S16. The ^{13}C NMR spectrum of compound **3** ($CDCl_3$, 125MHz)

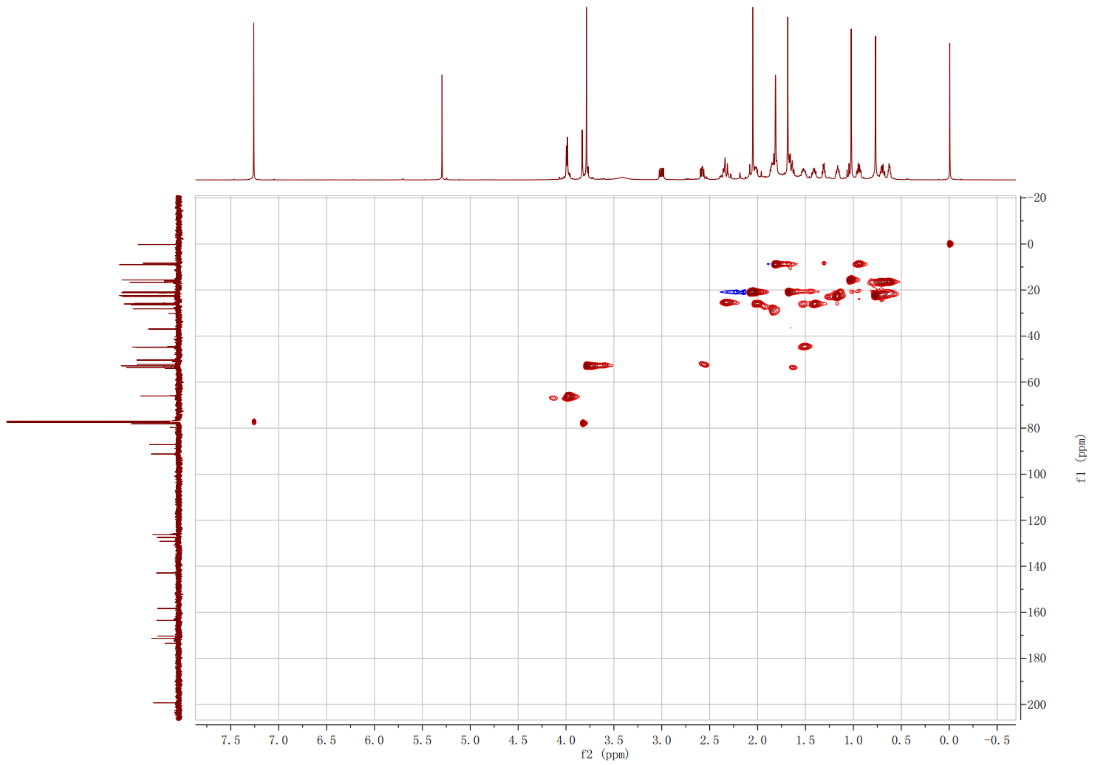

Figure S17. The HSQC spectrum of compound **3** in $CDCl_3$

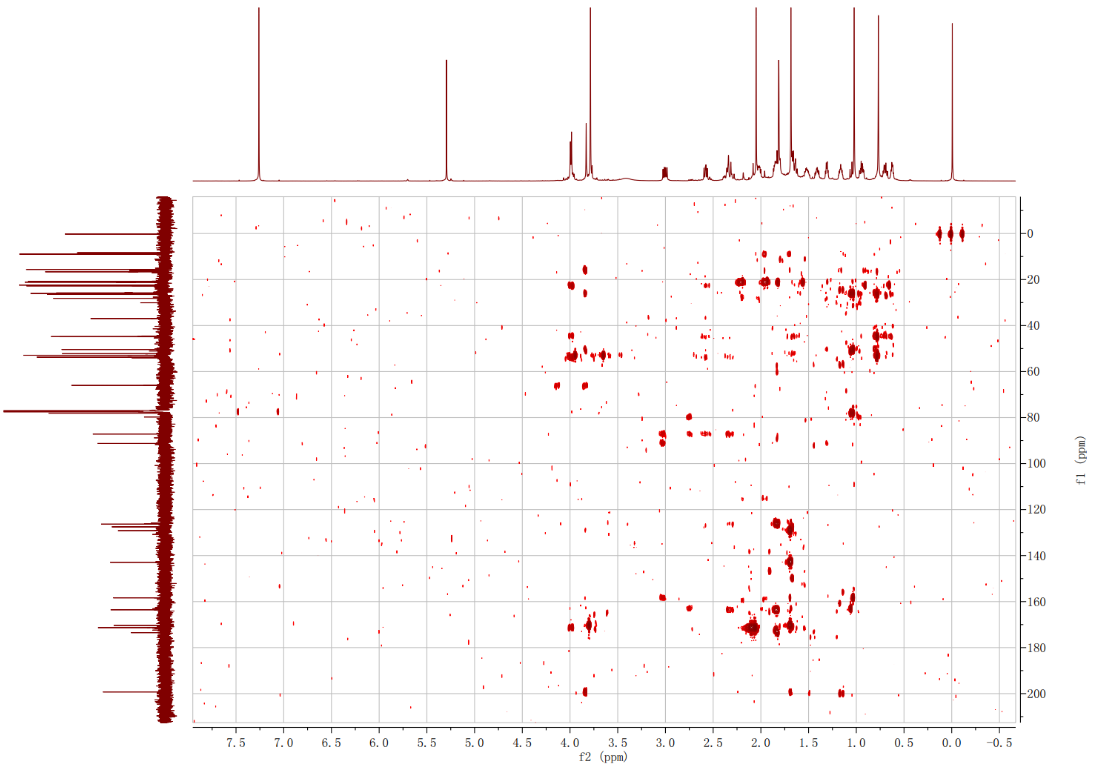

Figure S18. The HMBC spectrum of compound **3** in $CDCl_3$

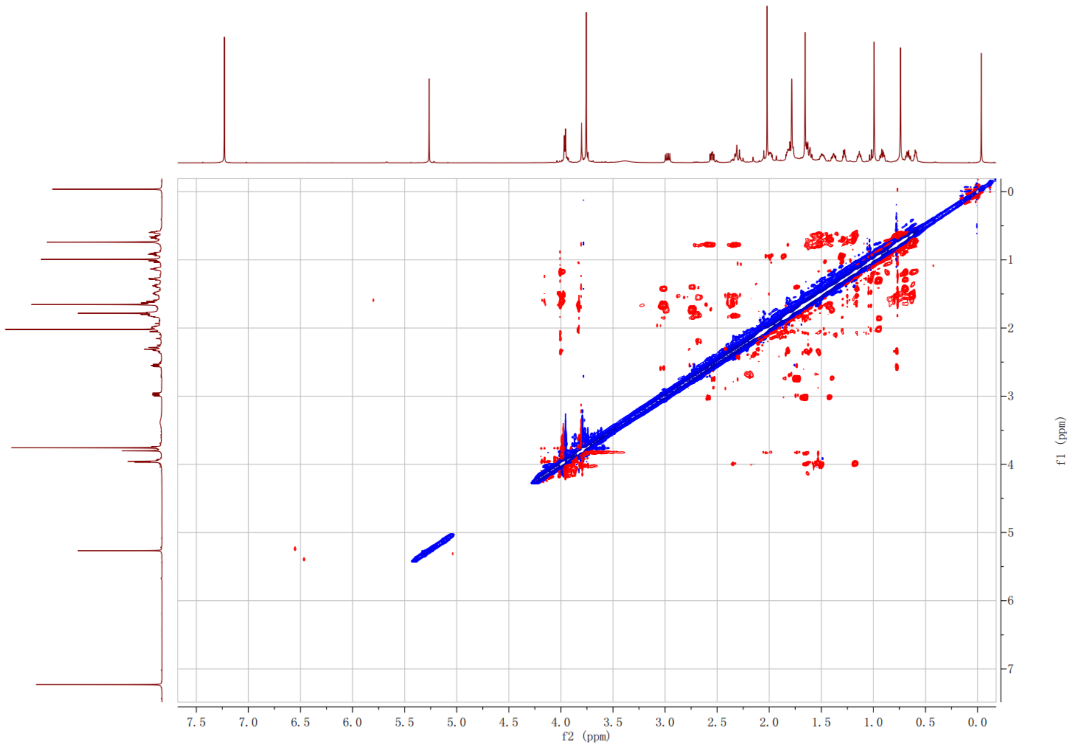

Figure S19. The ROESY spectrum of compound **3** in $CDCl_3$

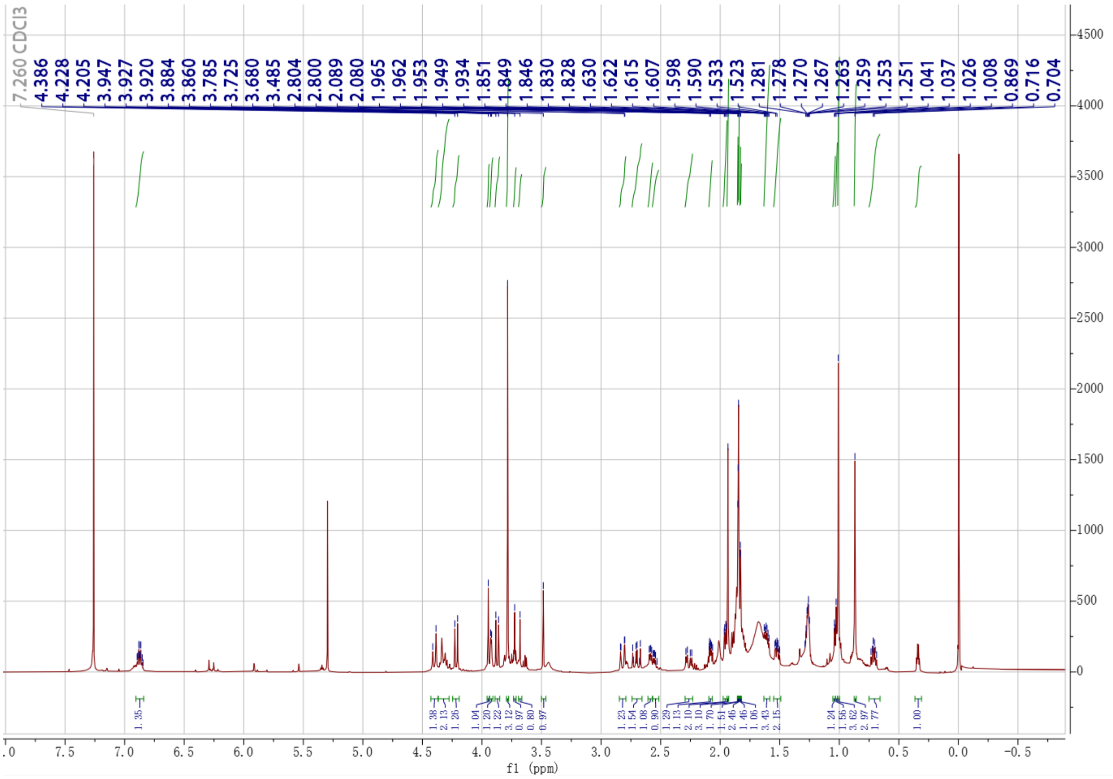

Figure S20. The ^{1}H NMR spectrum of compound **4** ($CHCl_3$, 500MHz)

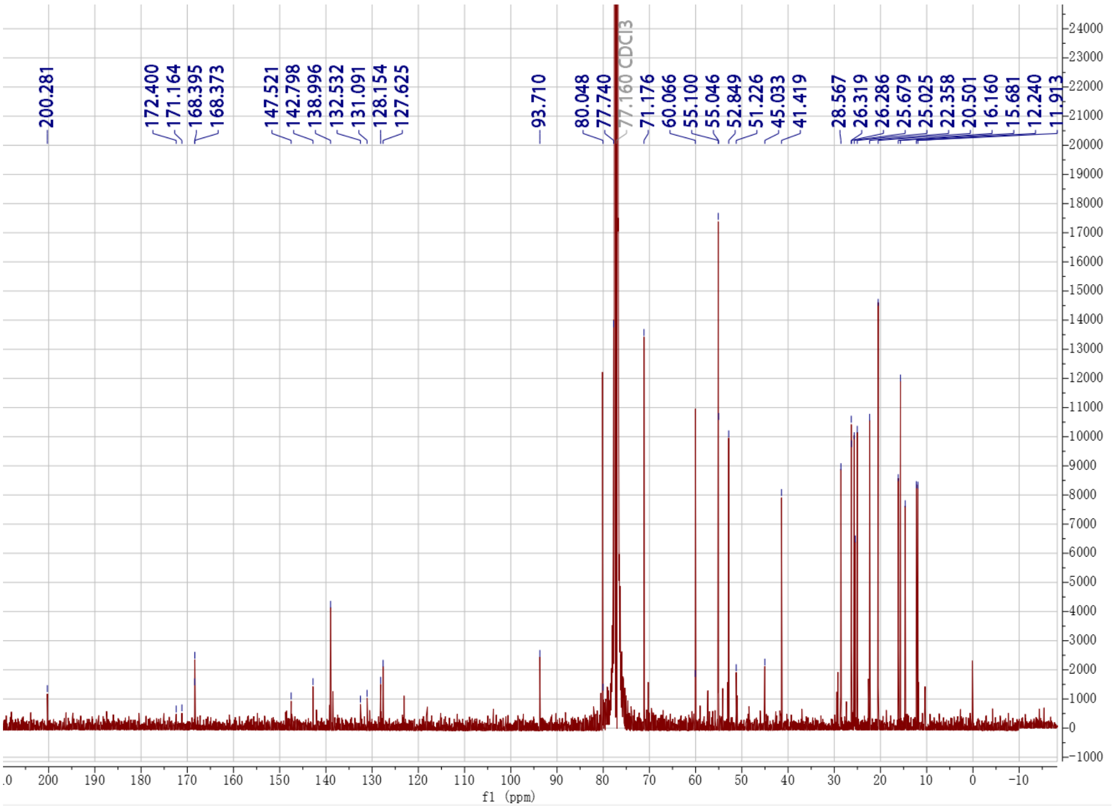

Figure S21. The ^{13}C NMR spectrum of compound **4** ($CHCl_3$, 125MHz)

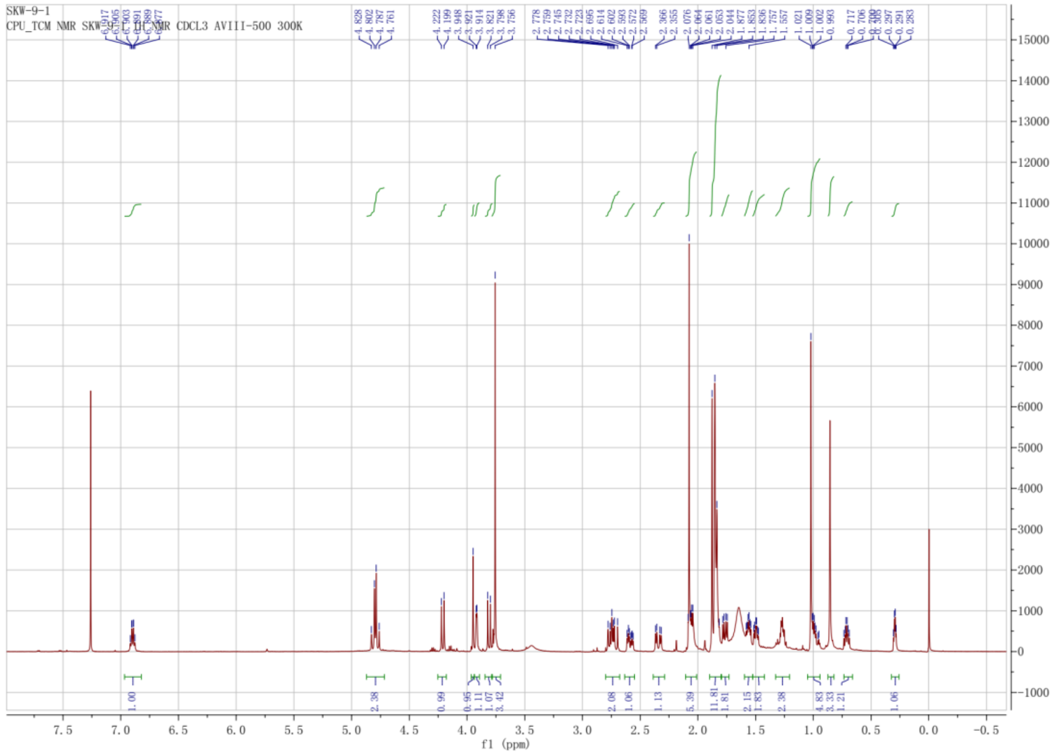

Figure S22. The ^{1}H NMR spectrum of compound **5** ($CHCl_3$, 500MHz)

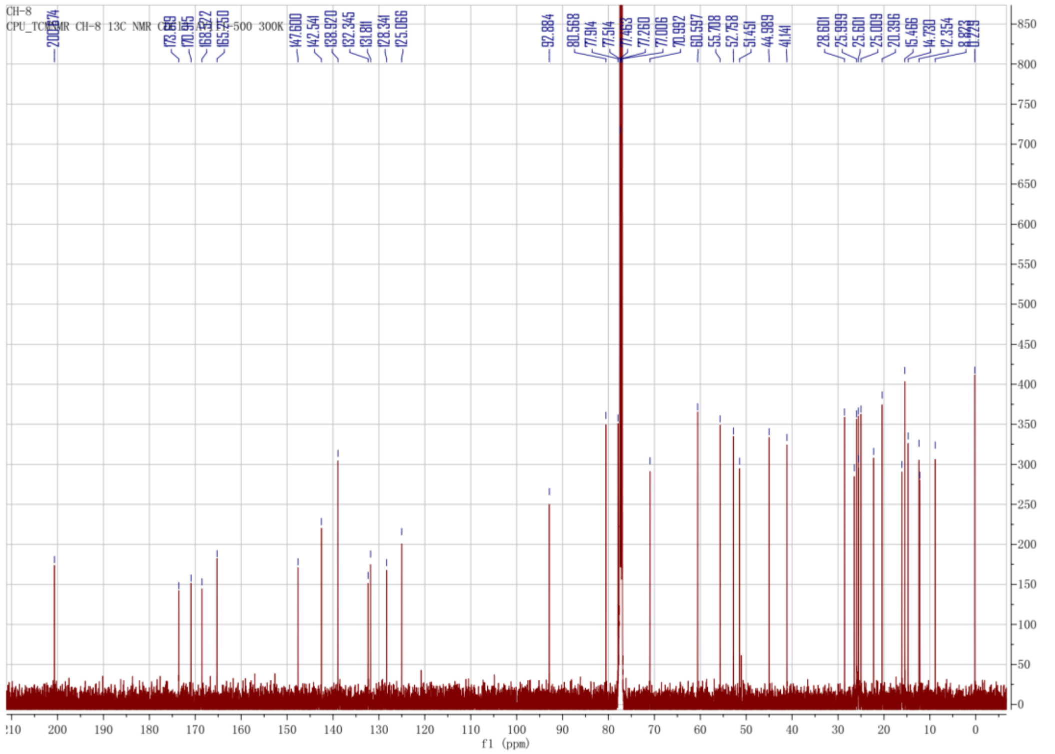

Figure S23. The ^{13}C NMR spectrum of compound **5** ($CHCl_3$, 125MHz)

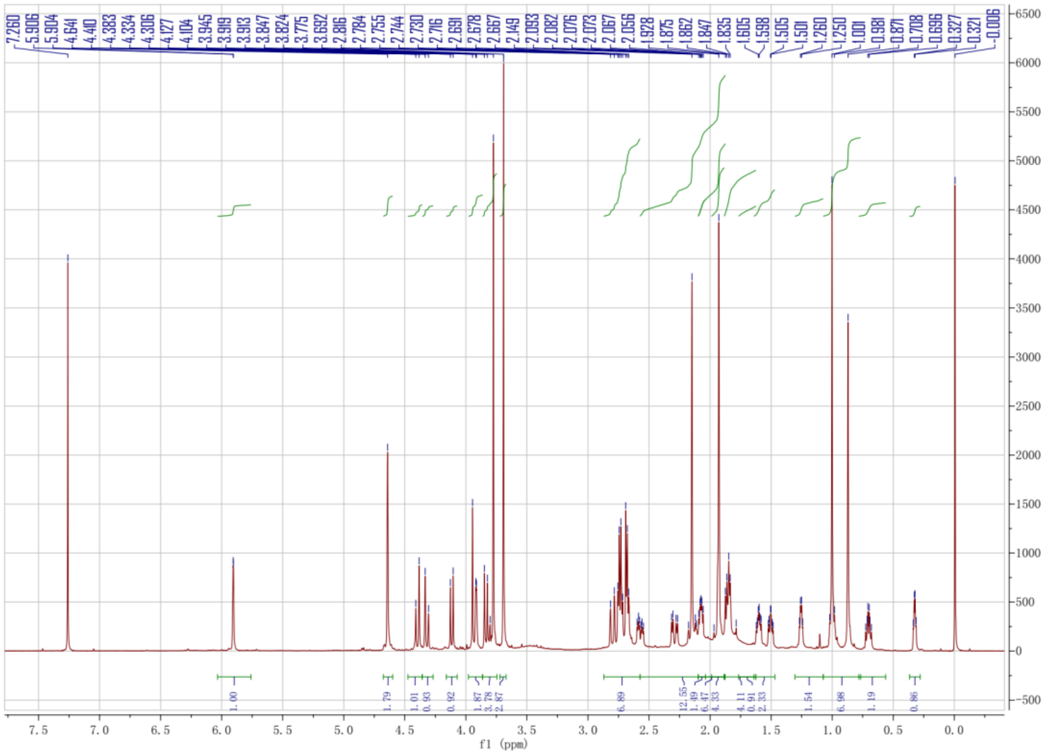

Figure S24. The ^{1}H NMR spectrum of compound **6** ($CHCl_3$, 500MHz)

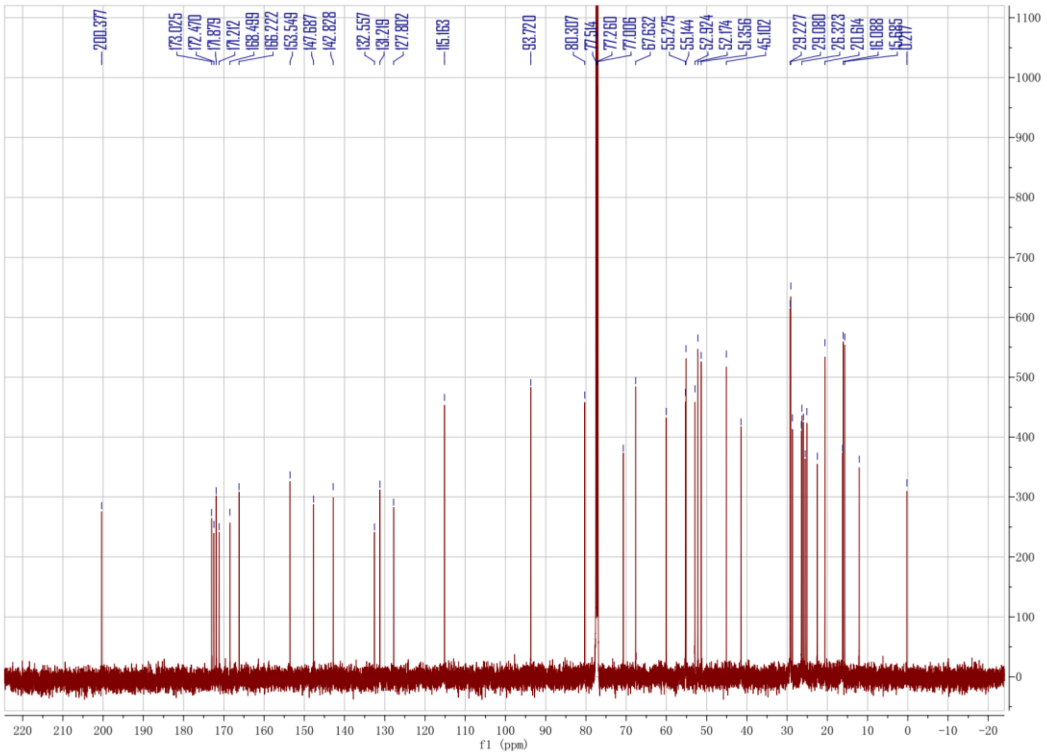

Figure S25. The ^{13}C NMR spectrum of compound **6** ($CHCl_3$, 125MHz)

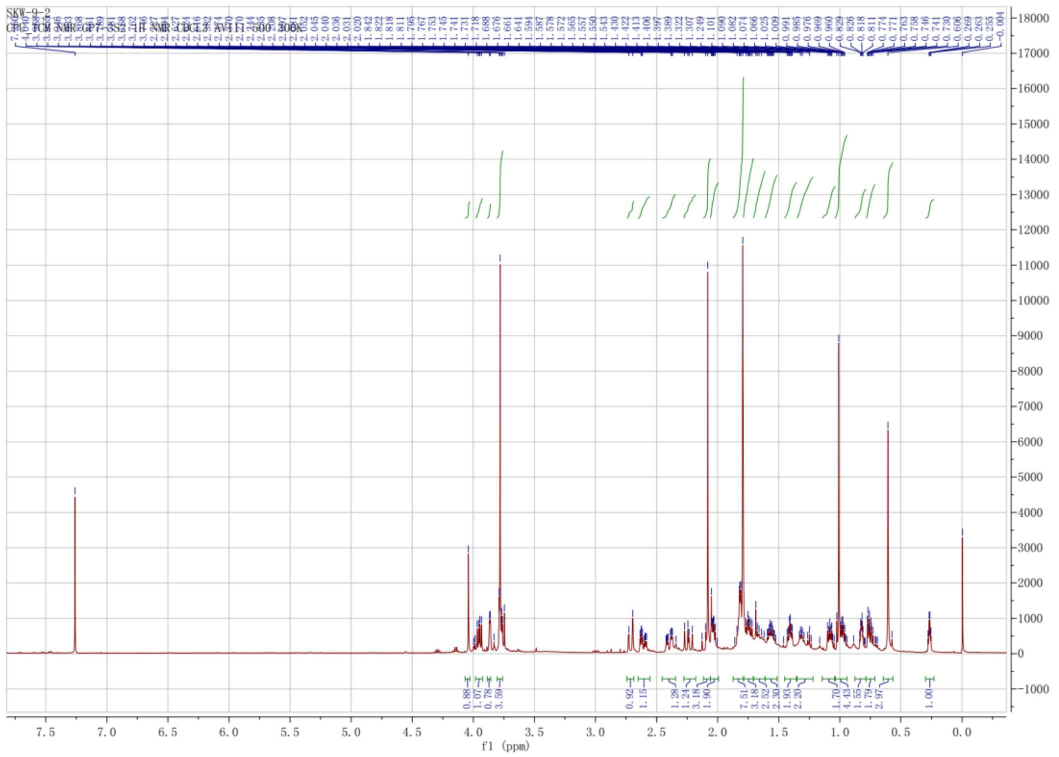

Figure S26. The ^{1}H NMR spectrum of compound **7** ($CHCl_3$, 500MHz)

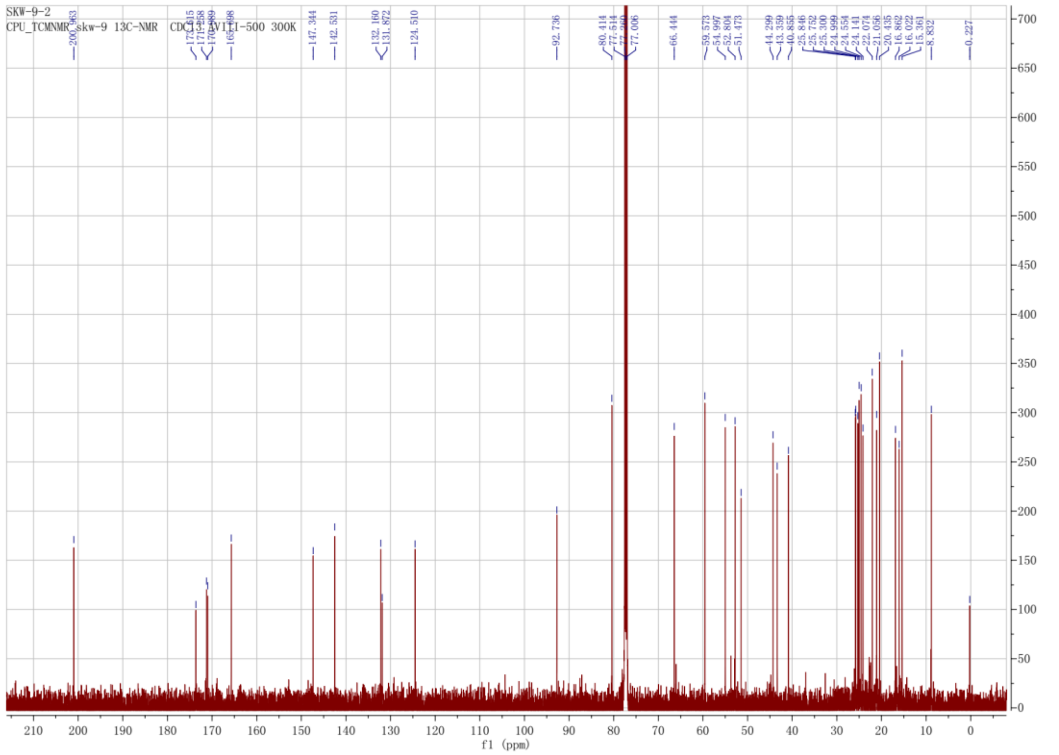

Figure S27. The ^{13}C NMR spectrum of compound **7** ($CHCl_3$, 125MHz)

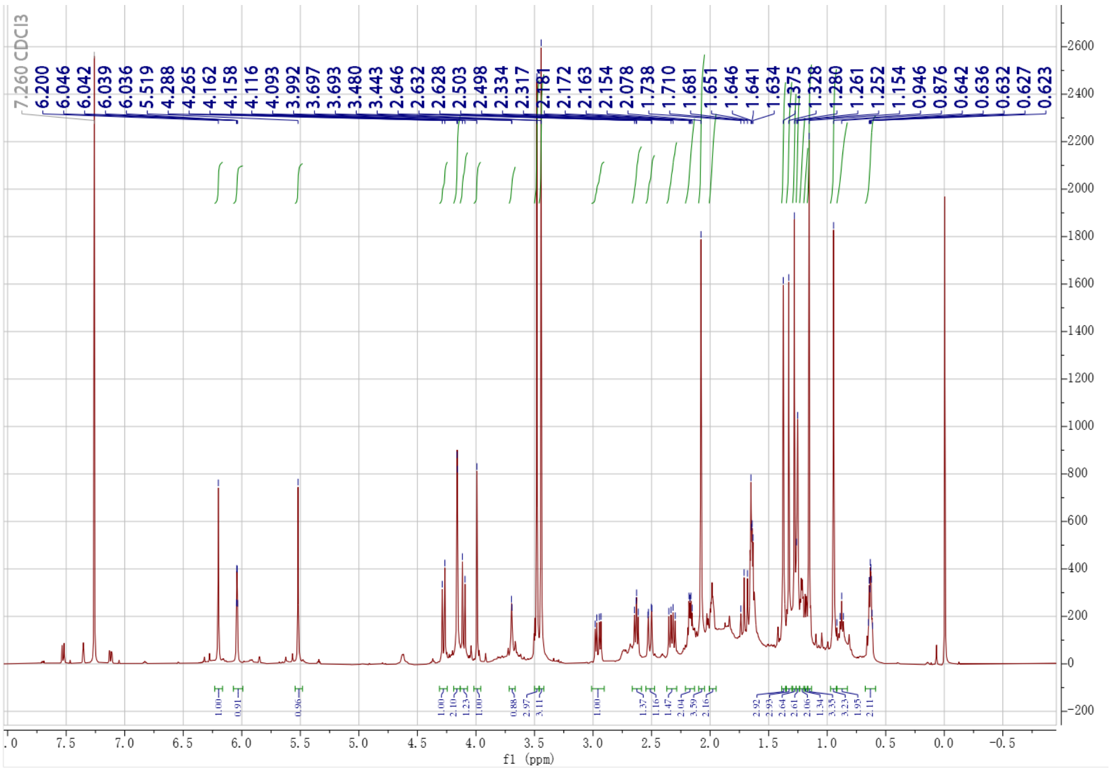

Figure S28. The ^{1}H NMR spectrum of compound **8** ($CHCl_3$, 500MHz)

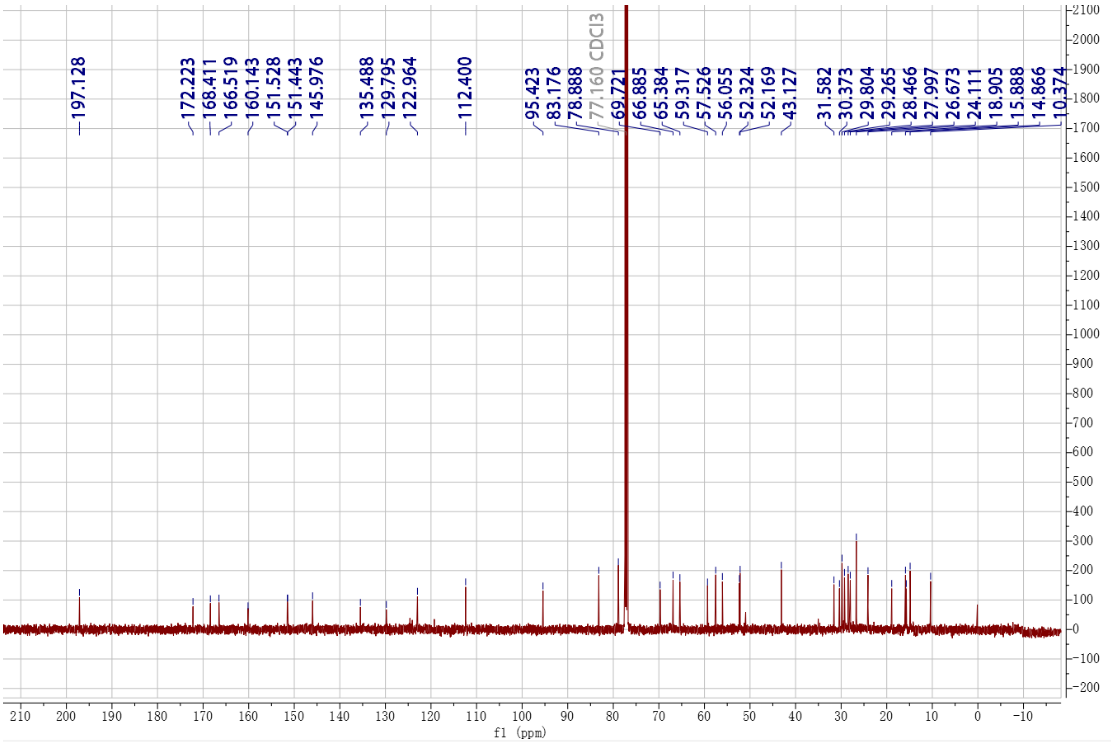

Figure S29. The ^{13}C NMR spectrum of compound **8** ($CHCl_3$, 125MHz)

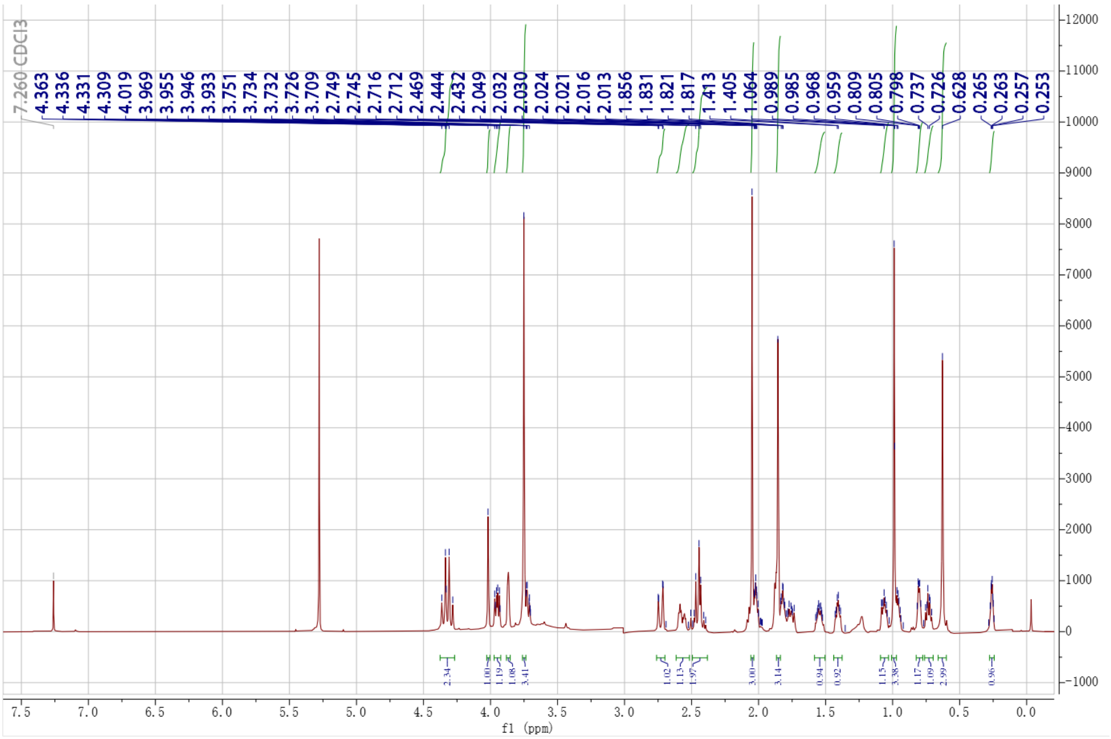

Figure S30. The ^{1}H NMR spectrum of compound **9** ($CHCl_3$, 500MHz)

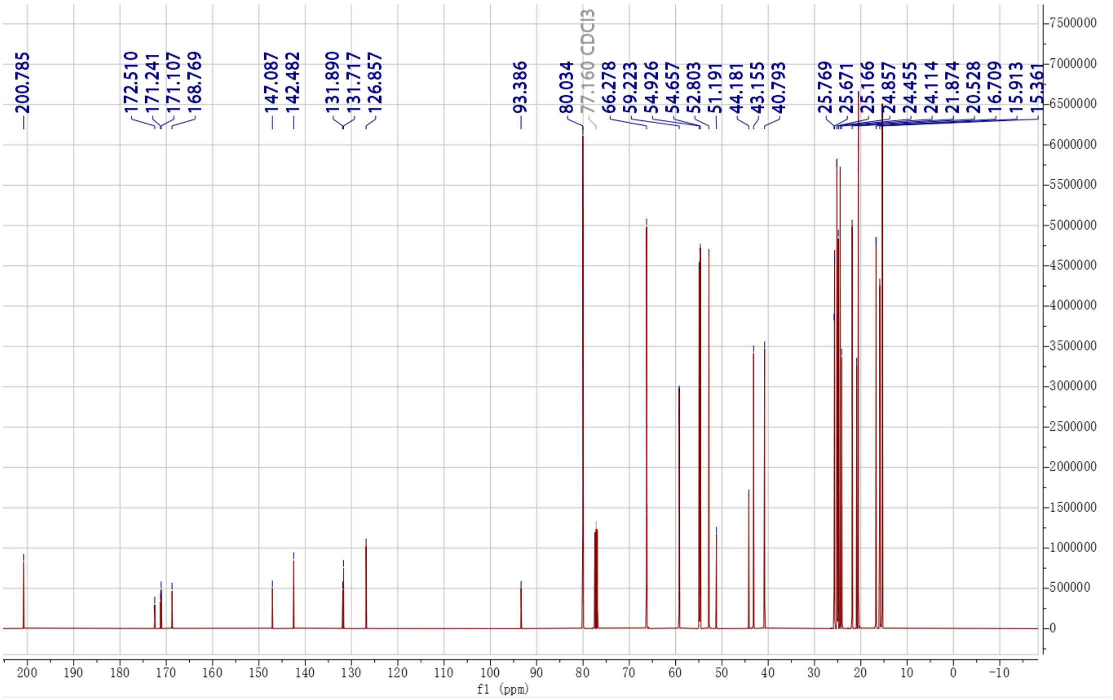

Figure S31. The ^{13}C NMR spectrum of compound **9** ($CHCl_3$, 125MHz)

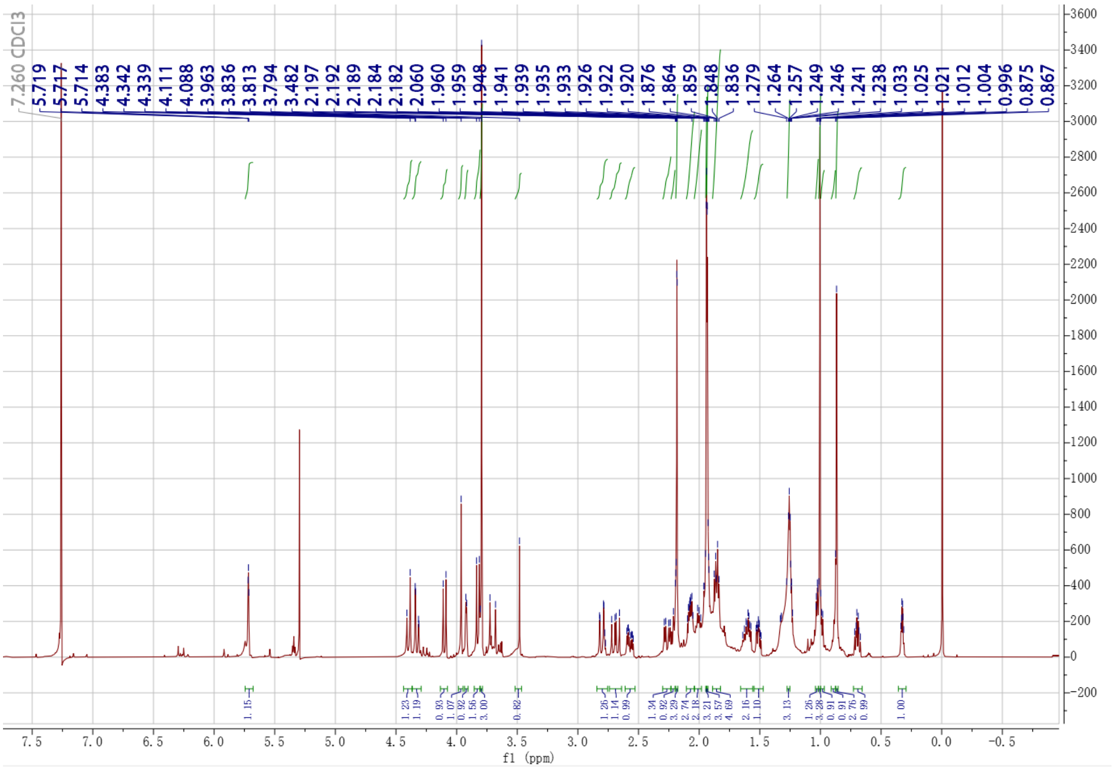

Figure S32. The ^{1}H NMR spectrum of compound **10** ($CHCl_3$, 500MHz)

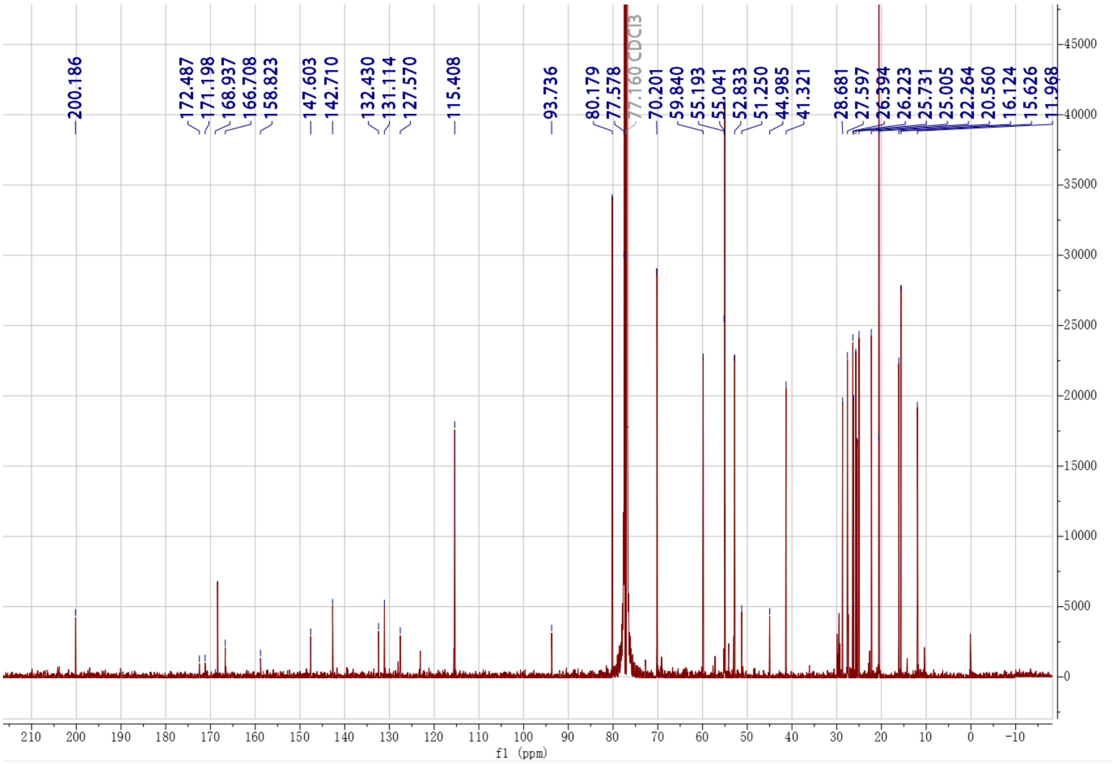

Figure S33. The ^{13}C NMR spectrum of compound **10** ($CHCl_3$, 125MHz)

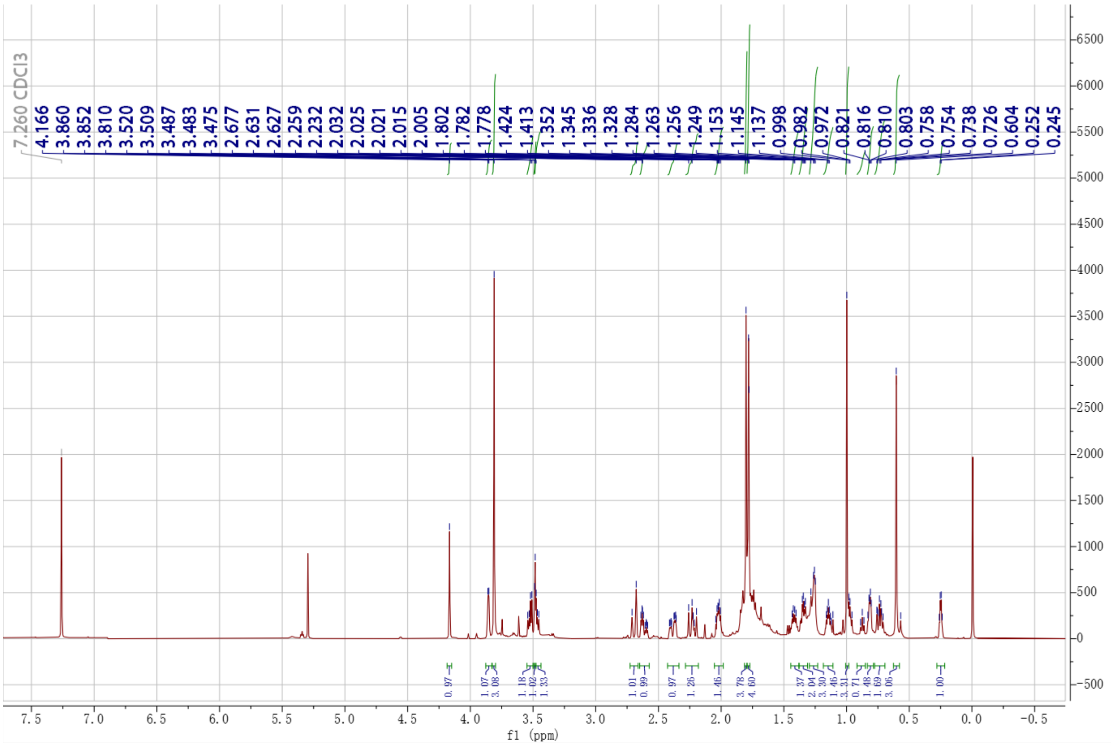

Figure S34. The ^{1}H NMR spectrum of compound **11** ($CHCl_3$, 500MHz)

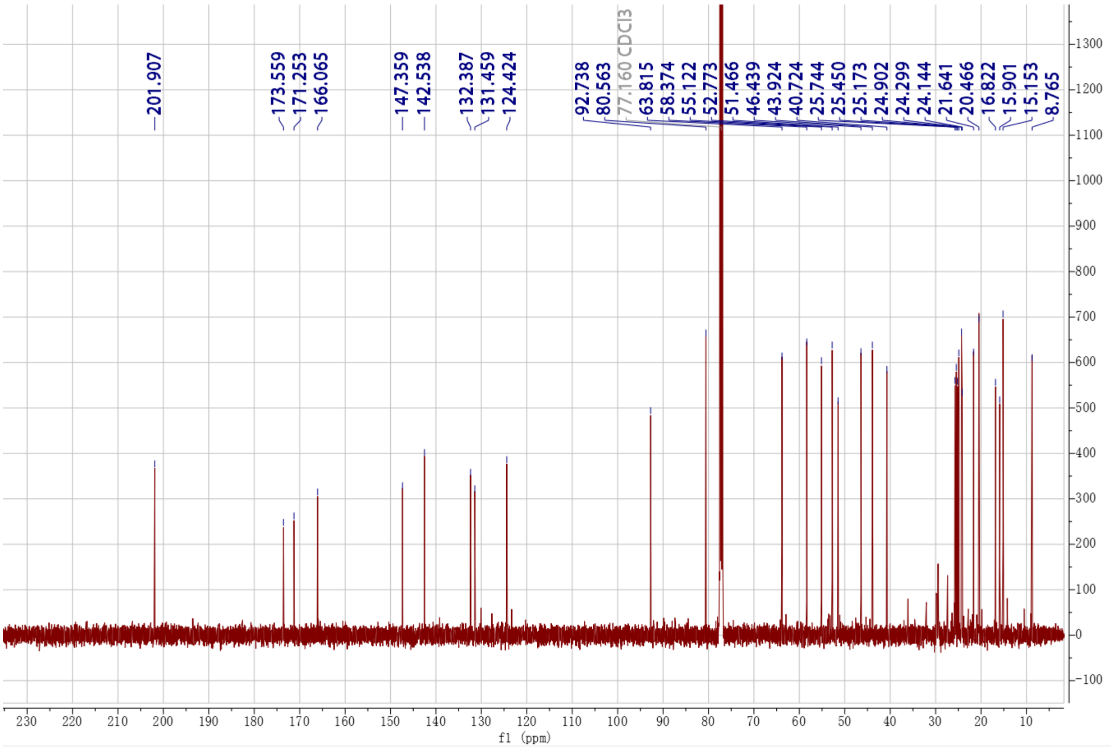

Figure S35. The ^{13}C NMR spectrum of compound **11** ($CHCl_3$, 125MHz)

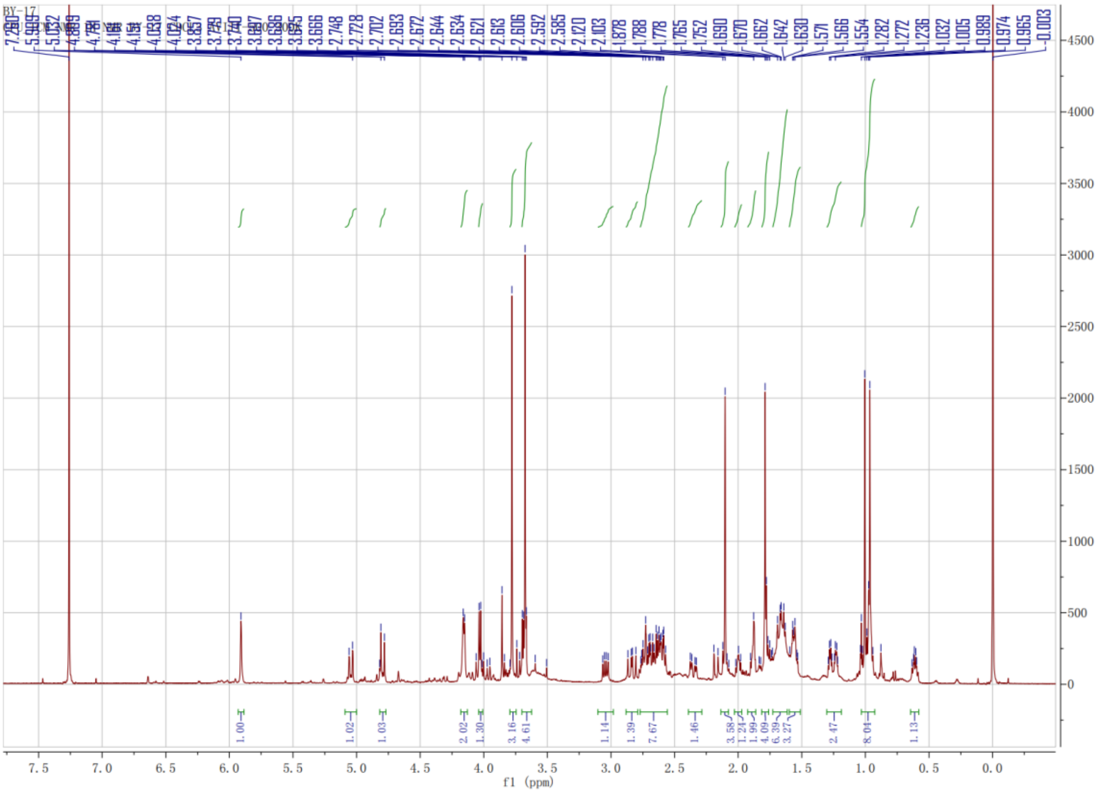

Figure S36. The ^{1}H NMR spectrum of compound **12** ($CHCl_3$, 500MHz)

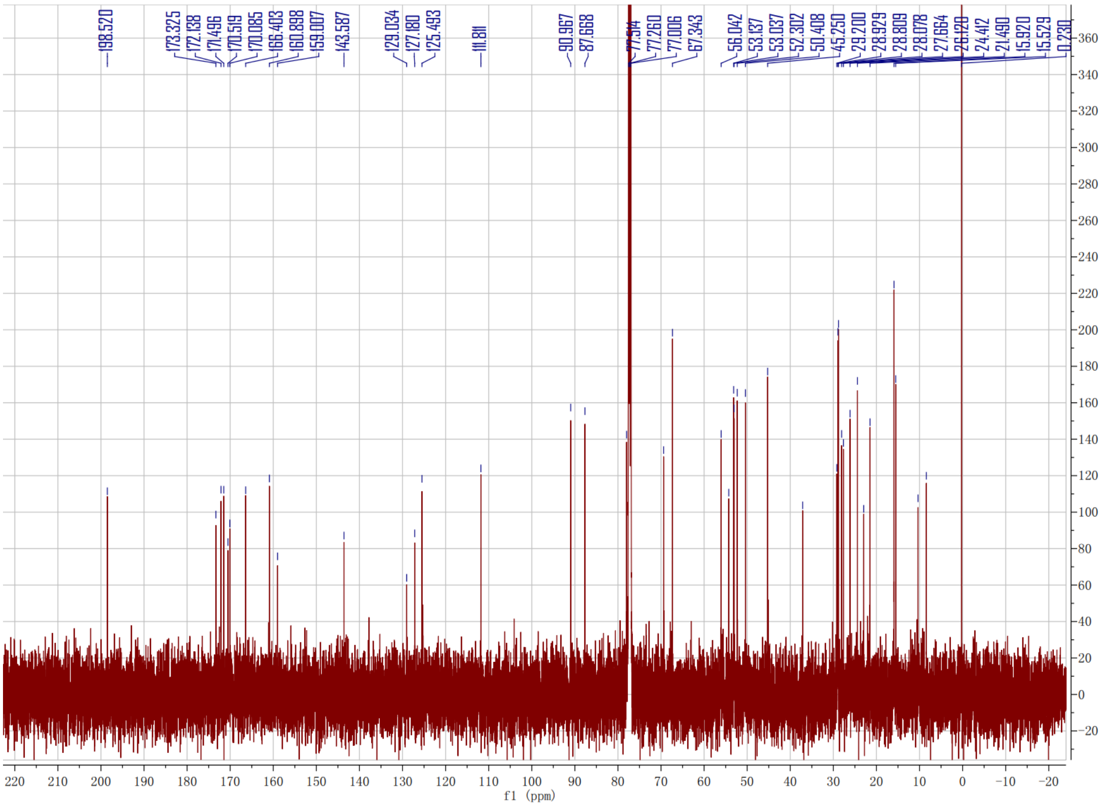

Figure S37. The ^{13}C NMR spectrum of compound **12** ($CHCl_3$, 125MHz)

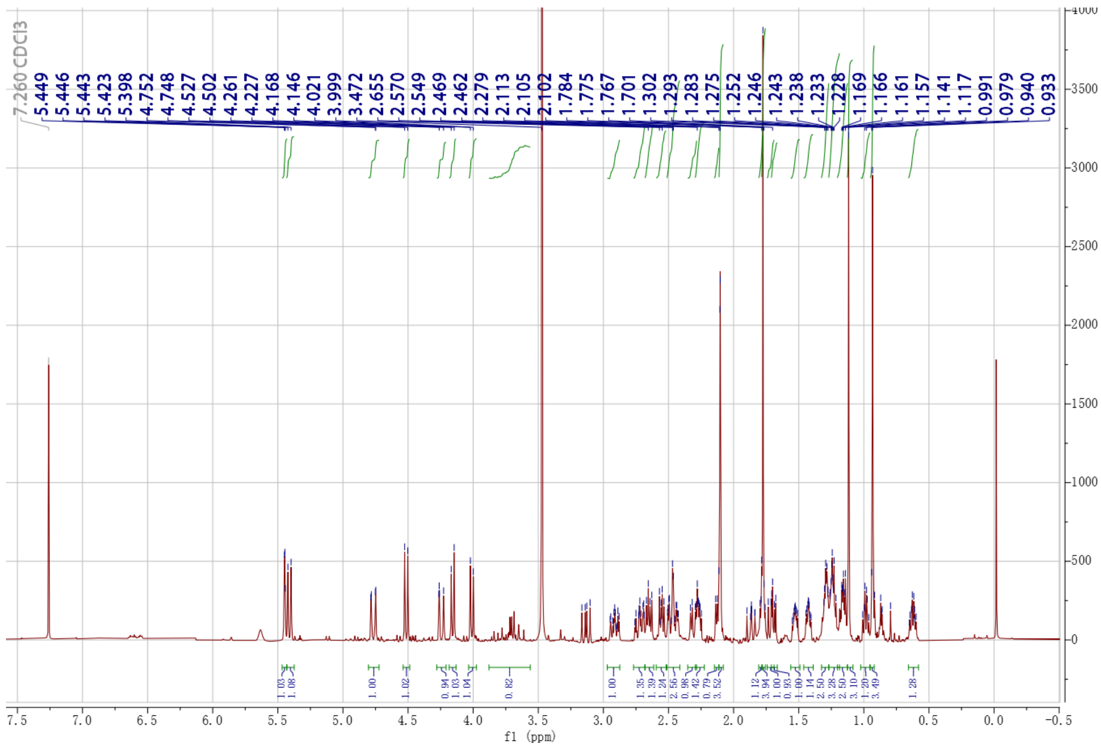

Figure S38. The ^{1}H NMR spectrum of compound **13** ($CHCl_3$, 500MHz)

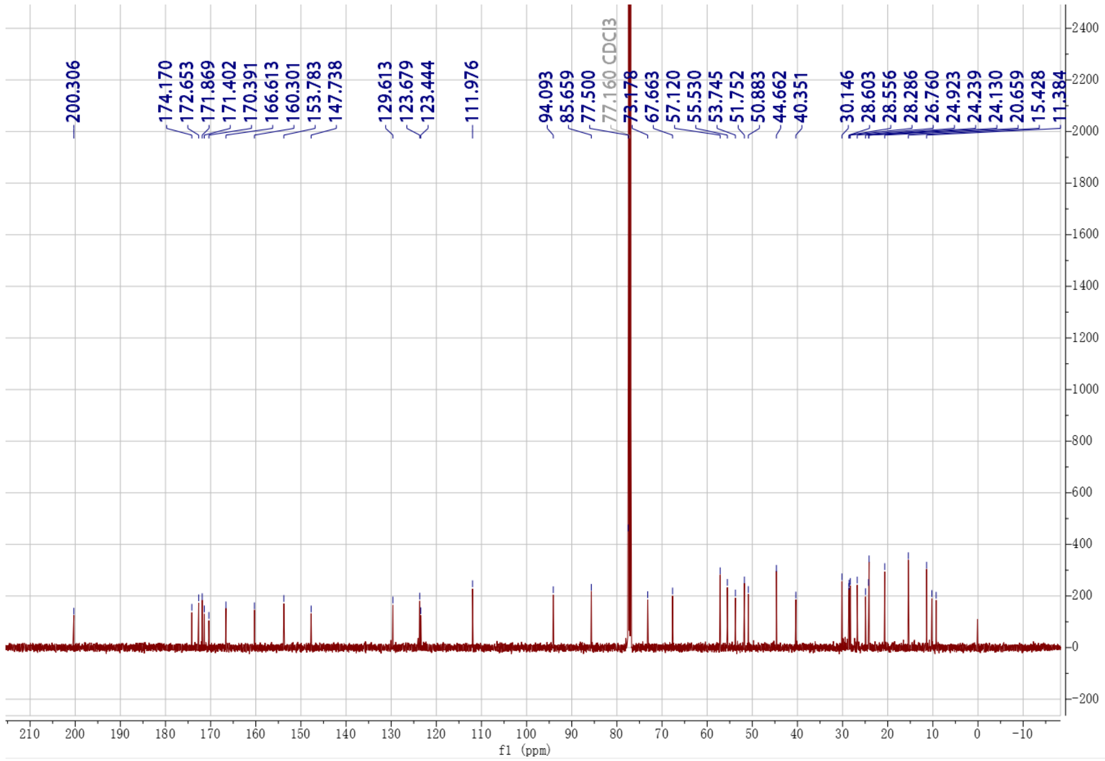

Figure S39. The ^{13}C NMR spectrum of compound **13** ($CHCl_3$, 125MHz)

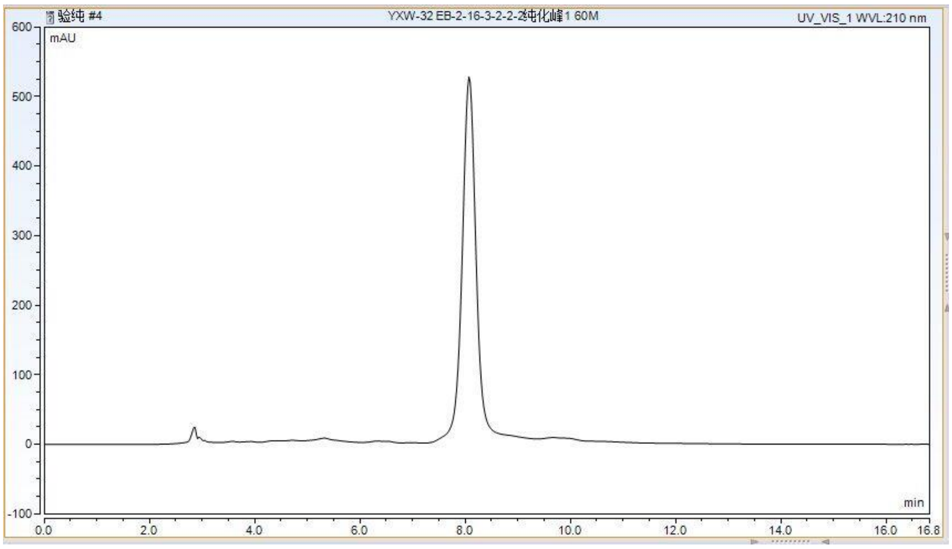

Figure S40. HPLC analysis of **8** after one month of the purification. (The chromatographic column specification is thermo scientific RP-C18 column (250 mm × 4.6 mm, 5.0 μm); column temperature of 30 ℃; flow rate of 1 mL/min; sample injection volume of 10 μL; retention time (t_R) of 8.1 min when eluted with MeOH-H_2O (60:40, v/v); detection wavelength of 210 nm.)

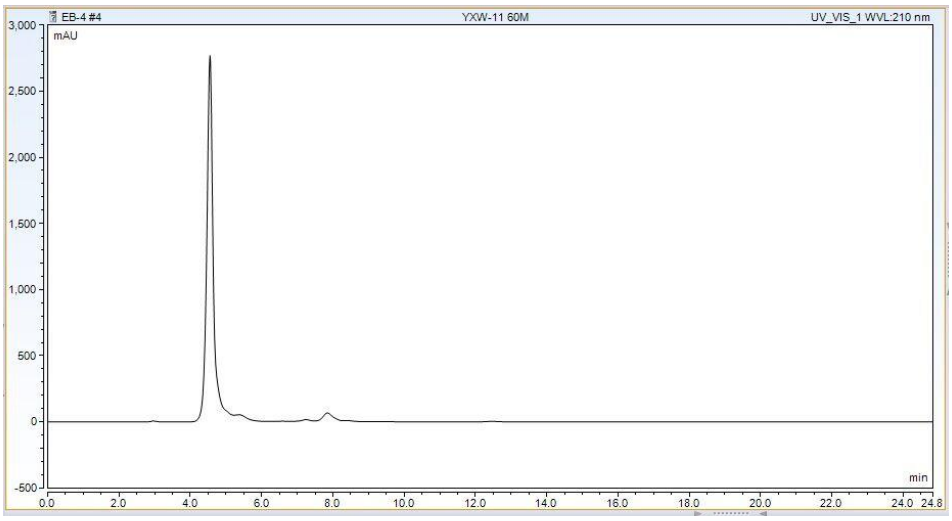

Figure S41. HPLC analysis of **13** after one month of the purification. (The chromatographic column specification is thermo scientific RP-C18 column (250 mm × 4.6 mm, 5.0 μm); column temperature of 30 ℃; flow rate of 1 mL/min; sample injection volume of 10.0 μL; retention time (t_R) of 4.7 min when eluted with MeOH-H_2O (60:40, v/v); detection wavelength of 210 nm.)

S1. ^{1}H and ^{13}C NMR Spectroscopic Data for Compounds **1**.

	**1**		**literature**	
No.	δ_H (mult)	δ_C	δ_H (mult)	δ_C
1	2.03, ddd (8.4, 6.2, 4.4)	26	2.03, ddd (8.4, 6.2, 4.4)	26
2	1.27, dt (5.6, 4.4)	8.1	1.27, dt (5.6, 4.4)	8.1
	0.97, m		0.97, m	
3	1.85, m	27.6	1.85, m	27.6
4		90.5		90.5
5		158.5		158.5
6		126.8		126.8
7		142.9		142.9
8		198.7		198.7
9	3.75, s	77.8	3.75, s	77.8
10		50.1		50.1
11		129		129
12		169.6		169.6
13	1.77, s	21	1.77, s	21
14	1.03, s	15.2	1.03, s	15.2
15	3.06, dd (14.2, 7.2)	36.7	3.06, dd (14.2, 7.2)	36.7
	1.64, m		1.64, m	
1′	1.60, m	27.3	1.60, m	27.3
2′	1.21, dt (5.6, 4.0)	10.3	1.21, dt (5.6, 4.0)	10.3
	0.62, dt (8.9, 5.6)		0.62, dt (8.9, 5.6)	
3′	1.61, m	29.2	1.61, m	29.2
4′		77.5		77.5
5′	1.62, m	54.8	1.62, m	54.8
6′	2.90, dd (17.8, 13.0)	22.4	2.90, dd (17.8, 13.0)	22.4
	2.28, dd (17.8, 6.6)		2.28, dd (17.8, 6.6)	
7′		170.2		170.2
8′		87.2		87.2
9′	2.61, dd (10.2, 7.2)	52.4	2.61, dd (10.2, 7.2)	52.4
10′		45.1		45.1
11′		125		125
12′		171.5		171.5
13′	4.86, d (13.0)	55.1	4.86, d (13.0)	55.1
	4.82, d (13.0)		4.82, d (13.0)	
14′	0.96, s	24	0.96, s	24
15′	4.22, d (11.4)	70.7	4.22, d (11.4)	70.7
	3.92, d (11.4)		3.92, d (11.4)	
1″		168.1		168.1
2″		127.9		127.9
3″	6.86, m	138.9	6.86, m	138.9
4″	1.85, d (1.0)	14.6	1.85, d (1.0)	14.6
5″	1.86, s	12.1	1.86, s	12.1
1‴		170.4		170.4
2‴	2.08, s	20.4	2.08, s	20.4
12-OMe	3.78, s	52.6	3.78, s	52.6

Data (^{1}H 500 MHz, ^{13}C 125 MHz) in $CDCl_3$. δ: ppm, J in Hz.

S2. ^{1}H and ^{13}C NMR Spectroscopic Data for Compounds **2**.

	**2**		**literature**	
No.	δ_H (mult)	δ_C	δ_H (mult)	δ_C
1	2.00, m	26.1	1.98, m	26.0
2	1.22, m	15.9	0.95, m	8.3
	1.01, m		1.24, m	
3	1.58, m	27.5	1.55, m	27.9
4		90.1		90.7
5		159.0		158.9
6		127.2		127.0
7		143.6		143.3
8		198.5		198.4
9	3.86, s	78.0	3.85, s	77.8
10		50.4		50.2
11		129.0		128.8
12		170.5		170.5
13	1.79, s	21.5	1.78, s	21.4
14	1.00, s	15.5	1.00, s	15.5
15	3.04, dd (14.4, 7.3)	37.1	3.06, dd (14.2, 7.1)	36.8
	1.67, overlapped		1.65, dd (14.2, 10.2)	
1′	1.88, m	27.7	1.87, m	27.5
2′	1.23, m	10.3	0.60, m	10.2
	0.61, m		1.22, m	
3′	1.56, m	28.1	1.55, m	29.0
4′		77.6		77.1
5′	1.76, m	54.3	1.76, m	54.0
6′	2.84, dd (13.5, 18.0)	22.9	2.34, dd (17.7, 6.9)	22.8
	2.35, dd (7.0, 18.0)		2.82, dd (17.7, 13.2)	
7′		170.1		170.3
8′		87.7		87.6
9′	2.59, m	53.1	2.58, dd (10.2, 7.1)	52.8
10′		45.3		45.0
11′		125.5		125.3
12′		171.5		171.6
13′	5.04, d (15.0)	56.0	5.06, dd (13.7, 2.0)	55.8
	4.79, d (15.0)		4.78, d (13.7)	
14′	0.97, s	24.4	0.96, s	24.3
15′	4.03, d (6.7)	69.4	4.02, s	69.1
1″		166.4		166.3
2″	5.91, s	113.8	5.90, dd (2.8, 1.5)	114.5
3″		160.9		160.9
4″	4.16, d (5.5)	67.3	4.16, m	67.1
5″	2.10, s	15.9	2.19, d (1.5)	15.8
1‴		172.1		173.3
2‴	2.64, m	28.8	2.64, m	28.6
3‴	2.70, m	28.9	2.70, m	28.7
4‴		173.3		172.1
12-OMe	3.79, s	53.0	3.77, s	53.1
1‴-OMe	3.67, s	52.3	3.67, s	52.3

Data (^{1}H 500 MHz, ^{13}C 125 MHz) in $CDCl_3$. δ: ppm, J in Hz.

S3. ^{1}H and ^{13}C NMR Spectroscopic Data for Compounds **3**.

	**3**		**literature**	
No.	δ_H (mult)	δ_C	δ_H (mult)	δ_C
1	2.03, m	26.1	2.03, m	26.1
2	1.31, m	8.4	1.31, m	8.4
	0.94, m		0.94, m	
3	1.41, m	26.8	1.41, m	26.8
4		91.2		91.2
5		158.4		158.4
6		127.5		127.5
7		142.9		142.9
8		199.3		199.3
9	3.83, s	78.1	3.83, s	78.1
10		50.4		50.4
11		129.2		129.2
12		170.3		170.3
13	1.69, s	21.1	1.69, s	21.1
14	1.02, s	15.6	1.02, s	15.6
15	3.00, dd (7.5, 14.0)	37.0	3.00, dd (7.5, 14.0)	37.0
	1.65, overlapped		1.65, overlapped	
1′	1.86, m	28.2	1.86, m	28.2
2′	0.62, m	16.6	0.62, m	16.6
	0.79, m		0.79, m	
3′	1.17, m	22.8	1.17, m	22.8
4′	1.52, m	44.8	1.52, m	44.8
5′	1.64, m	54.0	1.64, m	54.0
6′	2.32, m	25.8	2.32, m	25.8
7′		163.6		163.6
8′		87.2		87.2
9′	2.57, dd (7.5, 10.5)	52.2	2.57, dd (7.5, 10.5)	52.2
10′		44.9		44.9
11′		126.3		126.3
12′		173.5		173.5
13′	1.81, s	8.9	1.81, s	8.9
14′	0.77, s	22.4	0.77, s	22.4
15′	3.99, d (5.5)	66.0	3.99, d (5.5)	66.0
1″		171.3		171.3
2″	2.09, s	20.8	2.05, s	20.8
12-OMe	3.75, s	52.9	3.76, s	52.9

Data (^{1}H 500 MHz, ^{13}C 125 MHz) in $CDCl_3$. δ: ppm, J in Hz.

S4.The ECD spectrum of compounds **1-6**

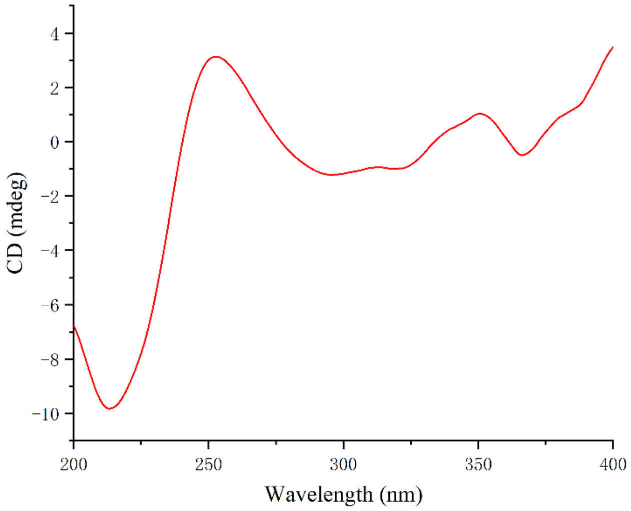

Compound 1

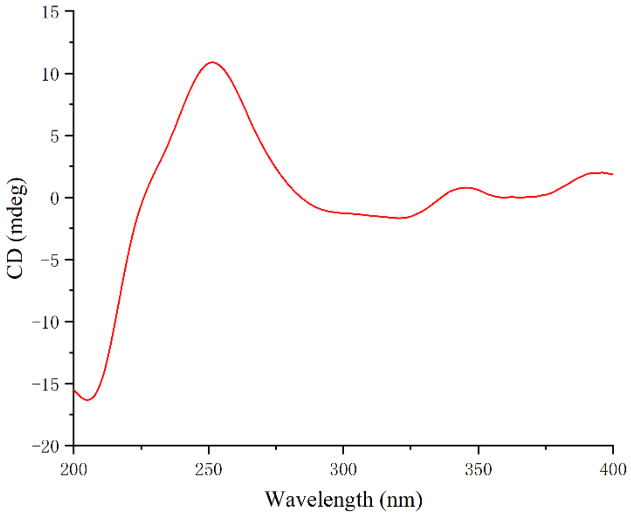

Compound 2

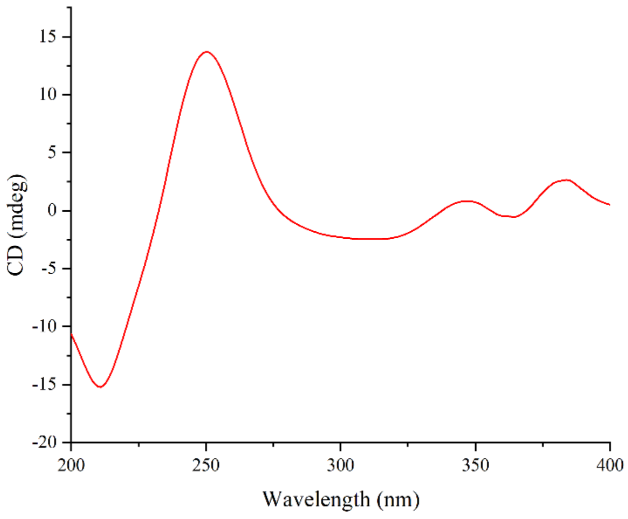

Compound 3

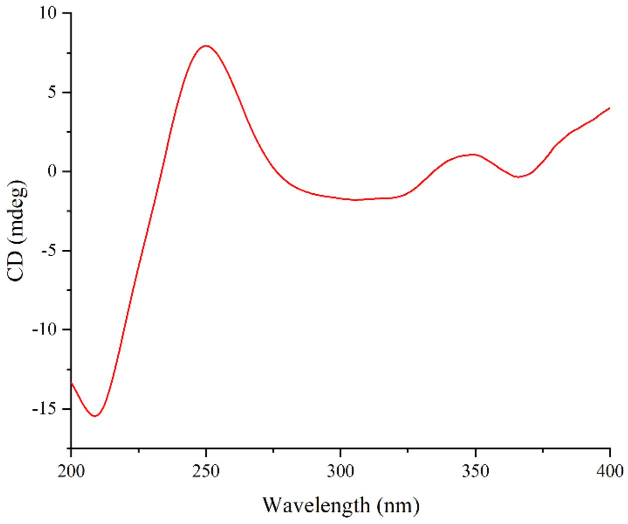

Compound 4

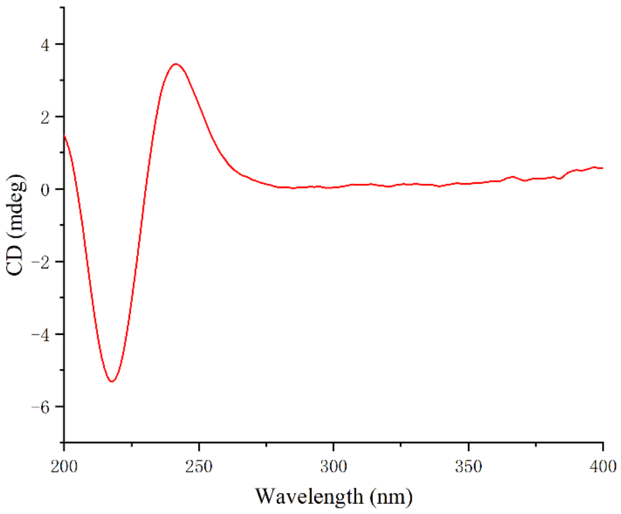

Compound 5

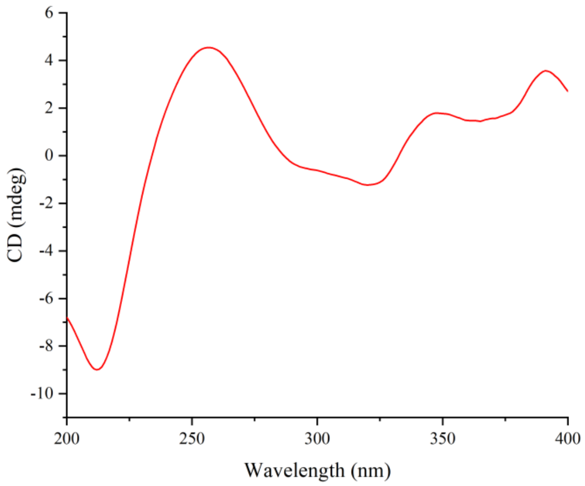

Compound 6

S5. The results of the DFT calculation

Path 1		unit	Path 2		
reactant	-1858.098	eV	reactant	-1858.098	eV
2 O·	-74.020	eV	2O·	-74.020	eV
product	-2006.808	eV	product	-2006.691	eV
transition state	-2005.778	eV	transition state	-2005.756	eV
Gibbs	-1759.027	KJ/mol	Gibbs	-1451.854	KJ/mol
TS	2704.176	KJ/mol	TS	2455.812	KJ/mol

Path 1		A1	EV	A2	EV
	LUMO	0.089	2.430	0.078	2.129
	HOMO	-0.314	-8.540	-0.340	-9.243

Path 2		A1	EV	A2	EV
	LUMO	0.089	2.286	0.078	2.129
	HOMO	-0.314	-8.540	-0.340	-9.243
	LUMO			0.048	1.300
	HOMO			-0.321	-8.737

S6. A1 Molecular coordinates used for Gaussian calculations

C	-3.30290000	2.12060000	-0.79230000
C	-1.93490000	2.67840000	-0.70260000
C	-1.92360000	4.10480000	-0.21180000
C	-0.71730000	1.80540000	-0.97580000
C	0.57550000	2.63970000	-0.83390000
C	0.49230000	4.18640000	-0.78030000
C	-0.86160000	4.89960000	-0.98170000
O	1.74130000	5.01220000	-0.59260000
C	-3.46590000	0.59910000	-0.73850000
C	-2.39340000	0.22640000	0.31640000
C	-2.47100000	-1.29790000	0.51080000
C	-2.04020000	-1.85150000	-0.85040000
C	-0.50910000	-1.83560000	-0.98230000
C	-0.00270000	-0.54700000	-0.30920000
C	-0.91690000	0.65860000	0.04740000
C	-3.93240000	-1.77780000	0.57770000
C	-4.11310000	-3.02540000	-0.33820000
C	-2.69840000	-3.23440000	-0.92590000

C	1.21070000	-0.54030000	0.58470000
C	1.01820000	0.60140000	1.54700000
O	-0.34930000	1.23960000	1.36810000
C	-1.64880000	-1.75170000	1.73230000
C	-4.13820000	-3.16530000	1.19460000
C	-1.93210000	-4.23260000	-0.03940000
C	2.42060000	-1.48870000	0.50480000
O	-2.64340000	-5.56310000	-0.07520000
C	-1.61910000	4.10040000	1.29690000
O	2.04350000	1.04680000	2.56140000
C	1.94600000	1.93130000	-0.81710000
C	3.10180000	2.46130000	0.05460000
C	2.14150000	0.63570000	-1.62590000
O	4.46540000	1.82090000	-0.04050000
O	2.86510000	3.57200000	1.04900000
C	5.49770000	2.89110000	-0.30150000
O	-1.20390000	4.88790000	-2.45200000
O	-2.72480000	-3.77200000	-2.33630000
C	-3.43200000	4.43720000	-0.47720000
C	-4.38220000	3.16280000	-0.47810000
C	-4.37120000	4.14130000	0.70120000
H	-2.73020000	0.75620000	1.26790000
H	-2.53130000	-1.17390000	-1.62430000
O	3.68290000	-0.71590000	0.80330000
H	-0.82020000	1.39630000	-2.03480000
H	-0.81650000	5.97450000	-0.60470000
H	-3.24010000	0.12080000	-1.74840000
H	-4.50330000	0.30260000	-0.37020000
H	-0.18180000	-1.94540000	-2.06860000
H	-0.09100000	-2.69980000	-0.36720000
H	-4.72090000	-1.15670000	1.11820000
H	-4.91810000	-2.59940000	-1.02370000
H	-1.70310000	-2.88130000	1.85560000
H	-2.07170000	-1.25700000	2.66810000
H	-0.56230000	-1.43400000	1.59810000
H	-5.18780000	-3.51550000	1.46870000
H	-3.24630000	-3.80630000	1.49760000
H	-1.90360000	-3.86030000	1.03420000
H	-0.86790000	-4.35140000	-0.43040000
H	2.48520000	-1.93100000	-0.54400000
H	2.29680000	-2.32780000	1.26620000
H	-3.67790000	-5.43200000	0.30570000
H	-2.37890000	3.44080000	1.83320000
H	-1.68460000	5.16420000	1.70090000

H	-0.56920000	3.68940000	1.46700000
H	2.76490000	-0.10250000	-1.02250000
H	1.12640000	0.17700000	-1.86650000
H	2.68350000	0.87970000	-2.59850000
H	5.25550000	3.42160000	-1.28100000
H	6.53070000	2.41410000	-0.37160000
H	5.48410000	3.64610000	0.55260000
H	-0.42410000	5.44020000	-3.01670000
H	-3.22620000	-4.76230000	-2.34150000
H	-3.67750000	4.83790000	-1.51590000
H	-5.13920000	3.38020000	-1.30230000
H	-3.87980000	3.71990000	1.63970000
H	-5.32530000	4.76020000	0.77930000
H	3.61580000	-0.27300000	1.81870000

S7. TS Molecular coordinates used for Gaussian calculations

C	-3.30290000	2.12060000	-0.79230000
C	-1.93490000	2.67840000	-0.70260000
C	-1.92360000	4.10480000	-0.21180000
C	-0.71730000	1.80540000	-0.97580000
C	0.57550000	2.63970000	-0.83390000
C	0.49230000	4.18640000	-0.78030000
C	-0.86160000	4.89960000	-0.98170000
O	1.74130000	5.01220000	-0.59260000
C	-3.46590000	0.59910000	-0.73850000
C	-2.39340000	0.22640000	0.31640000
C	-2.47100000	-1.29790000	0.51080000
C	-2.04020000	-1.85150000	-0.85040000
C	-0.50910000	-1.83560000	-0.98230000
C	-0.00270000	-0.54700000	-0.30920000
C	-0.91690000	0.65860000	0.04740000
C	-3.93240000	-1.77780000	0.57770000
C	-4.11310000	-3.02540000	-0.33820000
C	-2.69840000	-3.23440000	-0.92590000
C	1.21070000	-0.54030000	0.58470000
C	1.01820000	0.60140000	1.54700000
O	-0.34930000	1.23960000	1.36810000
C	-1.64880000	-1.75170000	1.73230000
C	-4.13820000	-3.16530000	1.19460000
C	-1.93210000	-4.23260000	-0.03940000
C	2.42060000	-1.48870000	0.50480000
O	-2.64340000	-5.56310000	-0.07520000
C	-1.61910000	4.10040000	1.29690000
O	2.04350000	1.04680000	2.56140000
C	1.94600000	1.93130000	-0.81710000
C	3.10180000	2.46130000	0.05460000
C	2.14150000	0.63570000	-1.62590000
O	4.46540000	1.82090000	-0.04050000
O	2.86510000	3.57200000	1.04900000
C	5.49770000	2.89110000	-0.30150000
O	-1.20390000	4.88790000	-2.45200000
O	-2.72480000	-3.77200000	-2.33630000
C	-3.43200000	4.43720000	-0.47720000
C	-4.38220000	3.16280000	-0.47810000
C	-4.37120000	4.14130000	0.70120000
H	-2.73020000	0.75620000	1.26790000
H	-2.53130000	-1.17390000	-1.62430000
O	3.68290000	-0.71590000	0.80330000
H	-0.82020000	1.39630000	-2.03480000

H	-0.81650000	5.97450000	-0.60470000
H	-3.24010000	0.12080000	-1.74840000
H	-4.50330000	0.30260000	-0.37020000
H	-0.18180000	-1.94540000	-2.06860000
H	-0.09100000	-2.69980000	-0.36720000
H	-4.72090000	-1.15670000	1.11820000
H	-4.91810000	-2.59940000	-1.02370000
H	-1.70310000	-2.88130000	1.85560000
H	-2.07170000	-1.25700000	2.66810000
H	-0.56230000	-1.43400000	1.59810000
H	-5.18780000	-3.51550000	1.46870000
H	-3.24630000	-3.80630000	1.49760000
H	-1.90360000	-3.86030000	1.03420000
H	-0.86790000	-4.35140000	-0.43040000
H	2.48520000	-1.93100000	-0.54400000
H	2.29680000	-2.32780000	1.26620000
H	-3.67790000	-5.43200000	0.30570000
H	-2.37890000	3.44080000	1.83320000
H	-1.68460000	5.16420000	1.70090000
H	-0.56920000	3.68940000	1.46700000
H	2.76490000	-0.10250000	-1.02250000
H	1.12640000	0.17700000	-1.86650000
H	2.68350000	0.87970000	-2.59850000
H	5.25550000	3.42160000	-1.28100000
H	6.53070000	2.41410000	-0.37160000
H	5.48410000	3.64610000	0.55260000
H	-0.42410000	5.44020000	-3.01670000
H	-3.22620000	-4.76230000	-2.34150000
H	-3.67750000	4.83790000	-1.51590000
H	-5.13920000	3.38020000	-1.30230000
H	-3.87980000	3.71990000	1.63970000
H	-5.32530000	4.76020000	0.77930000
H	3.61580000	-0.27300000	1.81870000

S8. A2 Molecular coordinates used for Gaussian calculations

C	-4.05070000	3.66800000	0.06870000
C	-3.33970000	2.30110000	0.22670000
C	-1.92060000	2.81600000	0.33830000
C	-1.76740000	4.31830000	0.12950000
C	-2.99200000	4.44660000	-0.80790000
C	-0.72250000	1.87090000	0.22710000
C	0.67310000	2.50450000	0.32820000
C	0.75270000	4.01070000	0.02900000
C	-0.47120000	4.67390000	-0.63340000
C	-4.48490000	4.76320000	-0.90540000
O	1.97400000	4.82220000	0.38810000
C	-3.39930000	1.17910000	-0.82500000
C	-2.44820000	0.09860000	-0.26480000
C	-2.72930000	-1.29390000	-0.85930000
C	-1.80280000	-2.37230000	-0.25230000
C	-1.09880000	-1.84800000	1.01760000
C	-0.26510000	-0.74860000	0.34300000
C	-0.94760000	0.45500000	-0.34270000
C	-4.12790000	-1.72010000	-0.36950000
C	-4.04610000	-2.98460000	0.50740000
C	-2.75040000	-3.57290000	-0.08180000
C	1.02170000	-1.06000000	-0.37550000
C	1.02320000	-0.19220000	-1.60800000
O	-0.29230000	0.56210000	-1.74280000
C	-2.65640000	-1.32200000	-2.39690000
C	-4.10490000	-1.59650000	1.16730000
C	-2.13640000	-4.70730000	0.75760000
C	2.08500000	-2.09760000	0.02890000
O	-3.13120000	-5.83840000	0.85980000
C	-1.88880000	5.09230000	1.45420000
O	2.16650000	-0.10460000	-2.59030000
C	1.91780000	1.69210000	0.74270000
C	3.31790000	2.09020000	0.23690000
C	1.76800000	0.48180000	1.68170000
O	4.53560000	1.35460000	0.74070000
O	3.47770000	3.19260000	-0.78160000
O	-0.31820000	6.17200000	-0.73820000
O	-3.09240000	-4.08830000	-1.45880000
O	3.45560000	-1.49300000	-0.16050000
C	5.76690000	2.16580000	0.41700000
O	-3.77640000	1.70400000	1.54250000
O	-5.18760000	1.27400000	1.42920000
H	-0.95500000	-2.63250000	-0.96880000

H	-2.69250000	-0.00140000	0.84420000
H	-4.00180000	4.17100000	1.09080000
H	-2.76720000	3.80630000	-1.72400000
H	-0.55200000	4.23170000	-1.68110000
H	-4.96390000	4.42480000	-1.88290000
H	-4.86480000	5.69890000	-0.37670000
H	-3.07500000	1.51430000	-1.86520000
H	-4.46750000	0.78180000	-0.85230000
H	-0.42720000	-2.65530000	1.46140000
H	-1.81800000	-1.43160000	1.79550000
H	-5.06370000	-1.13830000	-0.66190000
H	-5.01390000	-3.57350000	0.37990000
H	-2.90840000	-2.36880000	-2.77150000
H	-1.60130000	-1.04270000	-2.72640000
H	-3.40010000	-0.57280000	-2.82740000
H	-5.07460000	-1.59860000	1.76660000
H	-3.12590000	-1.49700000	1.73720000
H	-1.89070000	-4.31690000	1.80010000
H	-1.18370000	-5.08070000	0.25490000
H	1.98270000	-3.02700000	-0.62340000
H	1.94080000	-2.38010000	1.12400000
H	-4.06060000	-5.46410000	1.33750000
H	-2.86060000	4.79870000	1.97340000
H	-1.89390000	6.21170000	1.23850000
H	-1.00330000	4.83710000	2.12540000
H	2.27350000	-0.42800000	1.22070000
H	0.66210000	0.26800000	1.85420000
H	2.26430000	0.71940000	2.68000000
H	-1.20910000	6.59440000	-1.24810000
H	-3.82650000	-4.91610000	-1.36970000
H	3.54270000	-0.57830000	0.46270000
H	5.83840000	2.31230000	-0.71130000
H	6.69350000	1.61630000	0.78970000
H	5.69590000	3.18280000	0.92740000
H	-5.81580000	2.14900000	1.16100000

S9. B1/A1 Molecular coordinates used for Gaussian calculations

C	-4.05070000	3.66800000	0.06870000
C	-3.33970000	2.30110000	0.22670000
C	-1.92060000	2.81600000	0.33830000
C	-1.76740000	4.31830000	0.12950000
C	-2.99200000	4.44660000	-0.80790000
C	-0.72250000	1.87090000	0.22710000
C	0.67310000	2.50450000	0.32820000
C	0.75270000	4.01070000	0.02900000
C	-0.47120000	4.67390000	-0.63340000
C	-4.48490000	4.76320000	-0.90540000
O	1.97400000	4.82220000	0.38810000
C	-3.39930000	1.17910000	-0.82500000
C	-2.44820000	0.09860000	-0.26480000
C	-2.72930000	-1.29390000	-0.85930000
C	-1.80280000	-2.37230000	-0.25230000
C	-1.09880000	-1.84800000	1.01760000
C	-0.26510000	-0.74860000	0.34300000
C	-0.94760000	0.45500000	-0.34270000
C	-4.12790000	-1.72010000	-0.36950000
C	-4.04610000	-2.98460000	0.50740000
C	-2.75040000	-3.57290000	-0.08180000
C	1.02170000	-1.06000000	-0.37550000
C	1.02320000	-0.19220000	-1.60800000
O	-0.29230000	0.56210000	-1.74280000
C	-2.65640000	-1.32200000	-2.39690000
C	-4.10490000	-1.59650000	1.16730000
C	-2.13640000	-4.70730000	0.75760000
C	2.08500000	-2.09760000	0.02890000
O	-3.13120000	-5.83840000	0.85980000
C	-1.88880000	5.09230000	1.45420000
O	2.16650000	-0.10460000	-2.59030000
C	1.91780000	1.69210000	0.74270000
C	3.31790000	2.09020000	0.23690000
C	1.76800000	0.48180000	1.68170000
O	4.53560000	1.35460000	0.74070000
O	3.47770000	3.19260000	-0.78160000
O	-0.31820000	6.17200000	-0.73820000
O	-3.09240000	-4.08830000	-1.45880000
O	3.45560000	-1.49300000	-0.16050000
C	5.76690000	2.16580000	0.41700000
O	-3.77640000	1.70400000	1.54250000
O	-5.18760000	1.27400000	1.42920000
H	-0.95500000	-2.63250000	-0.96880000

H	-2.69250000	-0.00140000	0.84420000
H	-4.00180000	4.17100000	1.09080000
H	-2.76720000	3.80630000	-1.72400000
H	-0.55200000	4.23170000	-1.68110000
H	-4.96390000	4.42480000	-1.88290000
H	-4.86480000	5.69890000	-0.37670000
H	-3.07500000	1.51430000	-1.86520000
H	-4.46750000	0.78180000	-0.85230000
H	-0.42720000	-2.65530000	1.46140000
H	-1.81800000	-1.43160000	1.79550000
H	-5.06370000	-1.13830000	-0.66190000
H	-5.01390000	-3.57350000	0.37990000
H	-2.90840000	-2.36880000	-2.77150000
H	-1.60130000	-1.04270000	-2.72640000
H	-3.40010000	-0.57280000	-2.82740000
H	-5.07460000	-1.59860000	1.76660000
H	-3.12590000	-1.49700000	1.73720000
H	-1.89070000	-4.31690000	1.80010000
H	-1.18370000	-5.08070000	0.25490000
H	1.98270000	-3.02700000	-0.62340000
H	1.94080000	-2.38010000	1.12400000
H	-4.06060000	-5.46410000	1.33750000
H	-2.86060000	4.79870000	1.97340000
H	-1.89390000	6.21170000	1.23850000
H	-1.00330000	4.83710000	2.12540000
H	2.27350000	-0.42800000	1.22070000
H	0.66210000	0.26800000	1.85420000
H	2.26430000	0.71940000	2.68000000
H	-1.20910000	6.59440000	-1.24810000
H	-3.82650000	-4.91610000	-1.36970000
H	3.54270000	-0.57830000	0.46270000
H	5.83840000	2.31230000	-0.71130000
H	6.69350000	1.61630000	0.78970000
H	5.69590000	3.18280000	0.92740000
H	-5.81580000	2.14900000	1.16100000

S10. TS Molecular coordinates used for Gaussian calculations

C	-3.60740000	3.38730000	-1.38040000
C	-2.69480000	2.14600000	-1.19490000
C	-1.27250000	2.61820000	-1.55060000
C	-1.28750000	4.02650000	-0.93570000
C	-2.60170000	4.58430000	-1.49970000
C	-0.07610000	1.73470000	-1.11700000
C	0.74900000	2.53180000	-0.08080000
C	1.03330000	4.01240000	-0.40450000
C	0.01230000	4.78020000	-1.25940000
C	-3.76860000	4.63870000	-0.50760000
O	2.22890000	4.72640000	0.17870000
C	-2.63020000	1.55990000	0.22720000
C	-1.66940000	0.35370000	0.22190000
C	-2.33260000	-1.00930000	0.48390000
C	-1.36130000	-2.11920000	0.07390000
C	0.10990000	-1.85910000	0.43900000
C	0.36110000	-0.80980000	-0.66140000
C	-0.66190000	0.32150000	-0.94170000
C	-3.57380000	-1.48840000	-0.28600000
C	-3.55670000	-3.05070000	-0.09810000
C	-2.16280000	-3.34150000	0.51690000
C	1.00420000	-1.21040000	-1.96210000
C	0.10670000	-0.63030000	-3.02710000
O	-1.09590000	0.06600000	-2.40430000
C	-2.72180000	-0.94820000	1.97270000
C	-4.66700000	-2.19310000	0.52230000
C	-1.54110000	-4.62320000	-0.06700000
C	2.02640000	-2.34400000	-2.15780000
O	-2.38600000	-5.81410000	0.31540000
C	-1.43260000	4.09360000	0.59650000
O	0.30390000	-0.82970000	-4.51050000
C	1.28040000	1.88860000	1.22270000
C	1.76370000	2.75910000	2.40090000
C	1.31580000	0.36120000	1.38010000
O	2.43420000	2.10030000	3.58200000
O	1.51780000	4.24820000	2.41750000
O	-0.07830000	6.20980000	-0.78200000
O	-2.21330000	-3.47810000	2.01910000
O	3.41670000	-1.75670000	-2.20450000
C	3.62750000	1.31000000	3.10100000
O	-3.24130000	1.06050000	-2.08940000
H	-1.35790000	-2.15750000	-1.06570000
H	-1.01610000	0.49880000	1.14440000

H	-4.11160000	3.43200000	-2.40180000
H	-2.64590000	4.78270000	-2.62150000
H	0.62260000	1.66820000	-2.01520000
H	0.25490000	4.73700000	-2.37250000
H	-4.64500000	5.30480000	-0.80330000
H	-3.50560000	4.49230000	0.59180000
H	-3.65310000	1.25180000	0.62520000
H	-2.17980000	2.33710000	0.92140000
H	0.72740000	-2.78890000	0.20710000
H	0.26430000	-1.50520000	1.51150000
H	-3.53760000	-1.41250000	-1.42290000
H	-3.67670000	-3.38360000	-1.18180000
H	-3.23760000	-1.91240000	2.28950000
H	-3.44640000	-0.08180000	2.12710000
H	-1.78790000	-0.77810000	2.60370000
H	-5.68110000	-2.28420000	0.00950000
H	-4.60200000	-2.06860000	1.65370000
H	-0.48810000	-4.75450000	0.34990000
H	-1.49270000	-4.53740000	-1.20270000
H	1.80880000	-2.89140000	-3.13380000
H	1.95140000	-3.07380000	-1.28520000
H	-2.43410000	-5.89070000	1.42170000
H	-2.41610000	3.61270000	0.90990000
H	-1.44680000	5.18780000	0.91540000
H	-0.56060000	3.56510000	1.10550000
H	1.82050000	0.09260000	2.36660000
H	1.90380000	-0.09720000	0.51800000
H	0.24900000	-0.03870000	1.38070000
H	0.89610000	6.71600000	-0.94460000
H	-1.18460000	-3.61910000	2.41120000
H	3.62450000	-1.22100000	-1.25470000
H	4.36520000	2.01270000	2.58970000
H	3.28260000	0.51610000	2.35910000
H	4.13500000	0.80620000	3.98890000
O	-4.60220000	0.69580000	-1.63730000

S11. B2 Molecular coordinates used for Gaussian calculations

C	-4.04910000	3.67080000	0.06600000
C	-3.34010000	2.30360000	0.22920000
C	-1.92020000	2.81740000	0.34160000
C	-1.76570000	4.31880000	0.12840000
C	-2.98910000	4.44500000	-0.81100000
C	-0.72260000	1.87080000	0.23320000
C	0.67380000	2.50440000	0.33110000
C	0.75390000	4.00990000	0.03000000
C	-0.46970000	4.67260000	-0.63500000
C	-4.48150000	4.76230000	-0.91310000
O	1.97580000	4.82150000	0.38720000
C	-3.39890000	1.18080000	-0.82170000
C	-2.44970000	0.09990000	-0.26020000
C	-2.73190000	-1.29120000	-0.85610000
C	-1.80470000	-2.37010000	-0.25200000
C	-1.10450000	-1.85070000	1.02230000
C	-0.26790000	-0.75060000	0.35260000
C	-0.94790000	0.45440000	-0.33460000
C	-4.12990000	-1.72080000	-0.36700000
C	-4.04600000	-2.98810000	0.50590000
C	-2.75090000	-3.57270000	-0.08850000
C	1.02050000	-1.06210000	-0.36100000
C	1.02750000	-0.19350000	-1.59230000
O	-0.28820000	0.56010000	-1.73230000
C	-2.65920000	-1.31540000	-2.39330000
C	-4.10820000	-1.60210000	1.17000000
C	-2.13150000	-4.71210000	0.73950000
C	2.08390000	-2.09670000	0.04970000
O	-3.12040000	-5.84830000	0.83430000
C	-1.88860000	5.09740000	1.45040000
O	2.17560000	-0.10310000	-2.56870000
C	1.92020000	1.69250000	0.74090000
C	3.31660000	2.08880000	0.22380000
C	1.77620000	0.48390000	1.68290000
O	4.53810000	1.35390000	0.72020000
O	3.46920000	3.18980000	-0.79750000
O	-0.31560000	6.17000000	-0.74320000
O	-3.09520000	-4.07770000	-1.46920000
O	3.45510000	-1.50300000	-0.16790000
C	5.76450000	2.17880000	0.41080000
O	-4.11900000	2.59710000	-2.08540000
O	-4.03520000	4.04770000	-2.36670000
H	-0.95500000	-2.62570000	-0.96800000

H	-2.69590000	-0.00040000	0.84830000
H	-4.00190000	4.17670000	1.08660000
H	-2.76280000	3.80250000	-1.72500000
H	-0.54940000	4.22650000	-1.68130000
H	-4.90796932	4.38629376	-1.64655222
H	-4.86200000	5.70000000	-0.38870000
H	-3.07290000	1.51530000	-1.86140000
H	-4.46720000	0.78420000	-0.85030000
H	-0.43460000	-2.66080000	1.46350000
H	-1.82450000	-1.43640000	1.80060000
H	-5.06680000	-1.13990000	-0.65830000
H	-5.01310000	-3.57780000	0.37620000
H	-2.91150000	-2.36120000	-2.77080000
H	-1.60470000	-1.03460000	-2.72370000
H	-3.40390000	-0.56550000	-2.82070000
H	-5.07840000	-1.60770000	1.76850000
H	-3.13020000	-1.50070000	1.74110000
H	-1.88290000	-4.32970000	1.78430000
H	-1.17870000	-5.07710000	0.23140000
H	1.96830000	-3.03880000	-0.58200000
H	1.95240000	-2.35630000	1.15210000
H	-4.05060000	-5.48440000	1.31810000
H	-2.86110000	4.80560000	1.96930000
H	-1.89300000	6.21630000	1.23180000
H	-1.00410000	4.84380000	2.12360000
H	2.29200000	-0.42230000	1.22620000
H	0.67190000	0.26040000	1.85230000
H	2.26690000	0.72830000	2.68220000
H	-1.20540000	6.59300000	-1.25460000
H	-3.82890000	-4.90670000	-1.38430000
H	3.55280000	-0.57710000	0.43720000
H	5.83710000	2.34180000	-0.71540000
H	6.69440000	1.63060000	0.77750000
H	5.68490000	3.18770000	0.93570000
H	-5.10563068	4.51840624	-2.60644778
